# Antioxidant and Anti-Inflammatory Activities of *Silene dichotoma* Ehrh. subsp. *dichotoma*: Phytochemical Characterization and Metabolomic Insights into Oxidative Stress-Related Responses in HaCaT Cells

**DOI:** 10.3390/metabo16100765

**Published:** 2026-10-12

**Authors:** Melek Nur Bilal, Ozan Kaplan, Mustafa Çelebier, Vahap Murat Kutluay

**Affiliations:** 1 Department of Pharmacognosy, Faculty of Pharmacy, Hacettepe University, Sihhiye, Ankara 06100, Türkiye; melekbilal@hacettepe.edu.tr; 2 Department of Analytical Chemistry, Faculty of Pharmacy, Hacettepe University, Sihhiye, Ankara 06100, Türkiye; ozankaplan@hacettepe.edu.tr (O.K.); celebier@hacettepe.edu.tr (M.Ç.)

**Keywords:** *Silene dichotoma*, Caryophyllaceae, free radical scavenging, oxidative stress, HaCaT cells, metabolomics

## Abstract

Background/Objectives: *Silene dichotoma* Ehrh. subsp. *dichotoma* is a relatively understudied member of a genus known for diverse secondary metabolites. This study integrated phytochemical characterization, biological activity assessment, and LC–MS-based cellular metabolomics to investigate its antioxidant, NO-inhibitory, and cytoprotective potential. Methods: An 80% ethanol extract of the aerial parts was partitioned into *n*-hexane, ethyl acetate, *n*-butanol, and aqueous fractions and evaluated for DPPH and superoxide radical-scavenging activities and total phenolic and flavonoid contents. The most active fraction was subjected to bioactivity-guided isolation, and fractions were assessed for cytotoxicity, LPS-induced nitric oxide inhibition, H_2_O_2_-induced cytoprotection, and untargeted LC–MS metabolomic profiling. Results: The ethyl acetate fraction exhibited the highest phenolic (51.84 mg GAE/g) and flavonoid (176.56 mg QE/g) contents and the strongest antioxidant activity, yielding isorhamnetin, isorhamnetin-3-O-β-D-galactopyranoside, and quercetin, reported for the first time from this species. None of the extract or fractions showed marked cytotoxicity toward RAW 264.7 macrophages or HaCaT keratinocytes. The ethyl acetate fraction most strongly inhibited LPS-induced nitric oxide production (IC_50_ = 118.34 µg/mL). In H_2_O_2_-challenged HaCaT cells, the *n*-hexane fraction modestly increased viability to 67.77% at 200 µg/mL; however, no statistically significant overall cytoprotective effect was demonstrated. Untargeted LC–MS profiling yielded 99 putatively annotated metabolites. After averaging analytical duplicates within biological replicates, 38 of 75 metabolites eligible for the overall seven-group analysis showed an FDR-adjusted difference. Exploratory pairwise comparisons suggested treatment-associated changes in energy-related, redox-associated, nucleotide, amino-acid, and lipid-associated features. Conclusions: These findings identify this species as a source of flavonoids with antioxidant and nitric oxide-inhibitory potential while demonstrating the value of metabolomics in characterizing cellular responses beyond viability endpoints.

## 1. Introduction

Natural products constitute a fundamental and sustainable source for the discovery and development of novel therapeutic agents. Among natural resources with a wide range of biological activities, plants represent one of the most important groups [1]. It has been reported that 65–80% of the population in developed countries relies on plant-based remedies as part of primary healthcare, and plants continue to play an important role in the discovery of new drug candidates [2].

The genus *Silene* L. belongs to Caryophyllaceae, a family comprising more than 80 genera and over 2600 species, is represented by more than 700 species worldwide, 148 of which occur in Türkiye [3,4,5]. Owing to its ethnomedicinal uses and pharmacological potential, *Silene* genus has attracted considerable phytochemical and pharmacological interest [6]. Ethnobotanical records indicate that various *Silene* species have been consumed as food and used in traditional medicine [7].

Phytochemical studies on the genus *Silene* have led to the isolation of various secondary metabolites, including terpenoids (phytoecdysteroids, triterpenoid saponins), phenolics (flavonoids, anthocyanidins, anthraquinones, lignans), alkaloids, organic acids, sterols, and fatty acids [7,8]. In vitro and in vivo studies have reported diverse biological activities for members of the genus including antimicrobial, antifungal, antioxidant, antitumor, and hepatoprotective effects [5,7,9]. Several classes of secondary metabolites identified in *Silene* species, including phenolic compounds, flavonoids, terpenes, fatty acids, and phytoecdysteroids, have been reported to possess antioxidant, anti-inflammatory, and adaptogenic properties. Certain compounds belonging to these classes have also been associated with hair-growth-promoting effects [10,11,12]. Among the biological activities reported for the *Silene* genus, antioxidant activity has attracted particular attention in recent years because oxidative stress plays a pivotal role in the development of numerous pathological conditions [13,14], including skin disorders [15,16] and conditions associated with impaired hair growth [10]. Nevertheless, studies investigating the phytochemical properties and biological activities of *Silene dichotoma* Ehrh. subsp. *dichotoma* remains limited, highlighting the need for a comprehensive evaluation of its phytochemical and biological properties.

Oxidative stress arises from an imbalance between the formation of reactive oxygen species (ROS) and cellular antioxidant defenses. This imbalance may cause oxidative damage to cellular macromolecules, including lipids, proteins, and DNA, thereby contributing to the development of various pathological conditions [13,14]. Increasing evidence suggests that oxidative stress and impaired antioxidant defense play important roles in the pathogenesis of skin disorders [15]. Although the skin possesses effective defense mechanisms against oxidative stress, excessive accumulation of ROS may induce cellular damage, including apoptosis, in skin cells such as keratinocytes [16]. Oxidative stress is also closely associated with inflammatory signaling, while excessive nitric oxide (NO) production by activated macrophages represents an important feature of inflammatory responses [17].

Beyond conventional endpoint bioactivity assays, a more comprehensive understanding of how a plant extract modulates cellular oxidative stress responses requires insight into the underlying metabolic changes it induces. Metabolomic approaches have become increasingly important for characterizing cellular metabolic responses to treatment with plant extracts and fractions [18]. Metabolomics enables the investigation of small-molecule metabolites in biological systems under defined experimental conditions, thereby allowing the characterization of cellular metabolic profiles and the identification of biochemical pathways associated with oxidative stress and treatment-related cellular responses [19,20].

Despite this broad phytochemical and pharmacological interest in the genus, *S. dichotoma* Ehrh. subsp. *dichotoma* remains comparatively understudied, and no previous study has integrated phytochemical characterization, cell-based bioactivity assessment, and untargeted metabolomic profiling for this species. The present study therefore aimed to isolate and characterize selected constituents from *S. dichotoma* Ehrh. subsp. *dichotoma*, and evaluate the antioxidant, anti-inflammatory, and cytoprotective activities of the extract and sub-fractions. DPPH and superoxide (SO) radical-scavenging assays were used to guide the phytochemical investigation, and selected constituents were subsequently isolated and structurally characterized. Anti-inflammatory activity was evaluated by measuring NO production in lipopolysaccharide (LPS)-stimulated RAW 264.7 macrophages. An oxidative damage model was established in the immortalized human keratinocyte cell line HaCaT using hydrogen peroxide (H_2_O_2_), and the protective effects of the extract and fractions were assessed. Finally, metabolomic analyses were conducted to characterize the metabolic alterations induced by the *n*-hexane and ethyl acetate fractions, chosen according to their assay-dependent responses, under basal and H_2_O_2_-induced oxidative stress conditions. Minoxidil was profiled in a separate treatment-only group as a descriptive comparator. These analyses explored metabolomic responses to fraction exposure and simultaneous H_2_O_2_ co-exposure.

## 2. Materials and Methods

### 2.1. Chemicals and Instruments

#### 2.1.1. Chemicals

Unless otherwise stated, all chemicals and solvents were of analytical grade. Methanol (MeOH), ethanol (EtOH), chloroform, petroleum ether, aluminum chloride (AlCl_3_), sodium carbonate (Na_2_CO_3_), *n*-hexane, ethyl acetate, *n*-butanol, acetonitrile, formic acid, and aluminum-backed silica gel 60 F_254_ TLC plates (0.2 mm), as well as silica gel 60 (70–230 mesh) and LiChroprep RP-18 (40–63 µm) for column chromatography, were obtained from Merck (Darmstadt, Germany). Polyamide, Sephadex LH-20, gallic acid, quercetin, 2,2-diphenyl-1-picrylhydrazyl (DPPH), nitroblue tetrazolium (NBT), 3-(4,5-dimethylthiazol-2-yl)-2,5-diphenyltetrazolium bromide (MTT), LPS, fetal bovine serum (FBS), Dulbecco’s Modified Eagle Medium (DMEM), phosphate-buffered saline (PBS), penicillin–streptomycin, L-glutamine, sodium dodecyl sulfate (SDS), dimethyl sulfoxide (DMSO), H_2_O_2_, minoxidil, sulfanilamide, *N*-(1-naphthyl)ethylenediamine dihydrochloride, folin ciocalteu reagent and phosphoric acid were purchased from Sigma-Aldrich (St. Louis, MO, USA).

#### 2.1.2. Instruments

Solvent removal was performed using Büchi R-200 and R-210 rotary evaporators (Büchi, Flawil, Switzerland), and samples were lyophilized using a Christ Alpha 1-2 LDplus freeze dryer (Martin Christ, Osterode am Harz, Germany). Preparative high-performance liquid chromatography (HPLC) separations were carried out using a Shimadzu LC-40D system equipped with a Kromasil 100-10 C18 column (250 × 10 mm) (Nouryon, Bohus, Sweden) and a photodiode array (PDA) detector (Shimadzu, Kyoto, Japan). ^1^H and ^13^C NMR spectra were recorded on a Bruker UltraShield liquid-state NMR spectrometer (9.4 T; 400 MHz for ^1^H and 100 MHz for ^13^C; (Bruker, Rheinstetten, Germany). LC/MS analyses were performed using an Agilent Technologies 6530 quadrupole time-of-flight (Q-TOF) LC/MS system (Santa Clara, CA, USA). Cell culture experiments were conducted in a laminar flow cabinet (Chemocell LRCx-UV, Teknomar, Ankara, Türkiye) and a CO_2_ incubator (Sanyo MCO-18AIC, Osaka, Japan). Absorbance measurements were performed using a BioTek µQuant microplate reader (MQX 200; BioTek Instruments, Winooski, VT, USA).

### 2.2. Plant Material

The aerial parts of *S. dichotoma* Ehrh. subsp. *dichotoma* were collected at the flowering stage in June 2023 from the Beytepe Campus of Hacettepe University, Çankaya, Ankara, Türkiye. Taxonomic identification was performed by Assistant Professor Dr. Z. Ceren Arıtuluk Aydın (Department of Pharmaceutical Botany, Faculty of Pharmacy, Hacettepe University, Ankara, Türkiye). A voucher specimen (HUEF 26009) was deposited in the Herbarium of the Faculty of Pharmacy, Hacettepe University. The plant material was shade-dried at room temperature and pulverized prior to extraction.

### 2.3. Extraction, Fractionation and Isolation

The powdered aerial parts of *S. dichotoma* (420 g) were macerated overnight with 3 L of 80% ethanol, followed by extraction at 40 °C for 8 h. This extraction procedure was repeated twice using fresh 80% ethanol (2 L each). The combined extracts were filtered and concentrated under reduced pressure at 40 °C to obtain the crude 80% ethanol extract (72.14 g; extraction yield: 17.18%). A portion of the crude extract was reserved and lyophilized. The remaining extract was suspended in distilled water and successively partitioned with *n*-hexane, ethyl acetate, *n*-butanol, and water (5 × each) to obtain the corresponding fractions. All fractions were concentrated to dryness under reduced pressure, re-dissolved in water, and lyophilized prior to further analyses.

Together with the total phenolic content, total flavonoid content, and biological activity results (see Section 3.2), the ethyl acetate fraction (1.28 g) was selected for further phytochemical investigation.

The ethyl acetate fraction was subjected to silica gel column chromatography (Kieselgel 60, 70–230 mesh) using a CH_2_Cl_2_:MeOH:H_2_O gradient elution system. Fraction Fr. 90–99 (185.1 mg), eluted with CH_2_Cl_2_:MeOH:H_2_O (82.5:17.5:1.75, *v*/*v*/*v*), was further purified by Sephadex LH-20 column chromatography using 100% methanol as the mobile phase. Based on their TLC profiles, subfractions Fr. 8–19 and Fr. 45–55 were combined (76.7 mg) and further fractionated by vacuum liquid chromatography (VLC) on LiChroprep RP-18 using 45% methanol as the eluent, yielding fractions Fr. 46–50 (6.7 mg) and Fr. 51–70 (34.8 mg).

An analytical HPLC method was initially developed and optimized before being transferred to preparative HPLC for compound isolation. Fractions Fr. 46–50 and Fr. 51–70 were separately subjected to preparative HPLC under identical chromatographic conditions using a Shimadzu LC-40D preparative HPLC system equipped with a Kromasil 100-10 C18 column (250 × 10 mm). The mobile phases consisted of water containing 0.1% phosphoric acid (solvent A) and acetonitrile containing 0.1% phosphoric acid (solvent B). Gradient elution was performed as follows: 0–4 min, 10% B; 4–15 min, 20% B; 15–24 min, 22% B; 24–27 min, 25% B; 27–30 min, 35% B; 30–37 min, 100% B; 37–40 min, 10% B (re-equilibration), at a flow rate of 2.5 mL/min and a column temperature of 23 °C. Eluting compounds were monitored using a photodiode array (PDA) detector over the wavelength range of 190–500 nm. Preparative purification of fraction Fr. 51–70 yielded compounds **1** (16.7 mg) and **2** (7.0 mg), whereas purification of fraction Fr. 46–50 afforded compound **3** (5.0 mg).

### 2.4. Determination of Total Phenolic Content

The total phenolic content of the extract and fractions was determined using the Folin–Ciocalteu colorimetric method, with gallic acid as the reference. A calibration curve was constructed using gallic acid at concentrations of 0, 50, 100, 200, 400, and 800 mg/L. The extract and fraction solutions were prepared in distilled water at a concentration of 2 mg/mL. Aliquots (10 µL) of each sample or gallic acid solution were transferred to a 96-well plate, followed by the addition of 150 µL of diluted Folin–Ciocalteu reagent (1:4, reagent:water, *v*/*v*). After 3 min, 50 µL of sodium carbonate solution (2:3, Na_2_CO_3_:water, *w*/*v*) was added, and the reaction mixture was incubated at room temperature for 2 h. The absorbance was measured at 725 nm. The total phenolic content was calculated from the gallic acid calibration curve and expressed as milligrams of gallic acid equivalents per gram of dry extract (mg GAE/g dry extract). All measurements were performed in triplicate [21].

### 2.5. Determination of Total Flavonoid Content

Total flavonoid content was determined spectrophotometrically based on the formation of flavonoid–aluminum chloride (AlCl_3_) complexes, using quercetin as the reference. A calibration curve was constructed using quercetin solutions at concentrations of 0, 25, 50, 100, and 200 mg/L prepared in methanol. The extract and fraction solutions were prepared in methanol at a concentration of 1 mg/mL (or 500 µg/mL). Aliquots (1 mL) of each sample or standard solution were transferred into a 24-well plate, followed by the addition of 1 mL of 2% (*w*/*v*) aqueous AlCl_3_ solution. A corresponding sample blank was prepared for each sample to correct for the intrinsic color of the extracts and fractions. After incubation at room temperature for 10 min, the absorbance was measured at 415 nm. The total flavonoid content was calculated from the quercetin calibration curve and expressed as milligrams of quercetin equivalents per gram of dry extract (mg QE/g dry extract). All measurements were performed in triplicate [22].

### 2.6. Determination of the DPPH Radical Scavenging Activity

The DPPH radical scavenging activity of the extract and fractions was determined using the stable free radical DPPH. The extract and fraction solutions were prepared in methanol at concentrations of 50, 100, 200, 400, and 800 µg/mL. Quercetin was selected as the positive reference compound in this cell-free assay based on its experimentally characterized reaction with DPPH radicals [23]. It provided a chemically defined antioxidant reference for comparing the extracts and fractions under the same assay conditions.

Aliquots (200 µL) of each sample or methanol (blank) were transferred into a 96-well plate, followed by the addition of 50 µL of 1 mM DPPH solution. The reaction mixtures were incubated in the dark at room temperature for 30 min, and the absorbance was measured at 517 nm. The percentage of DPPH radical scavenging activity was calculated according to the following equation:

(1) **% Inhibition = 100 × [(A_blank_ − A_sample_)/A_blank_]**

where A_blank_ is the absorbance of the blank and A_sample_ is the absorbance of the sample.

All measurements were performed in triplicate. The IC_50_ values were calculated by linear regression analysis of the concentration–response curves [24,25].

### 2.7. Determination of the SO Radical Scavenging Activity

SO radical scavenging activity of the extract and fractions was determined using the NBT reduction assay. SO radicals were generated in an alkaline DMSO system and reduced NBT to a colored formazan product. The extract and fraction solutions were prepared in DMSO at concentrations of 50, 100, 200, 400, and 800 µg/mL. Quercetin was selected as the positive reference compound in this cell-free assay based on its experimentally documented superoxide-scavenging activity [26]. It provided a chemically defined antioxidant reference for comparing the extracts and fractions under the same assay conditions.

Aliquots of 10 µL of 1 mg/mL NBT solution prepared in DMSO were added to a 96-well plate, followed by 30 µL of each sample, DMSO (blank), or standard solution, and 100 µL of alkaline DMSO (90 µL DMSO containing 10 µL of 5 mM NaOH). The absorbance was measured at 560 nm.

The percentage of superoxide radical scavenging activity was calculated according to the method of Hyland et al. [27] using the following equation:

(2) **% Inhibition = 100 × [(A_blank_ − A_sample_)/A_blank_]**

where A_blank_ represents the absorbance of the reaction mixture in the absence of the extract/fraction (NBT and alkaline DMSO only), and A_sample_ represents the absorbance of the well containing the extract, fraction, or positive control.

All measurements were performed in triplicate. The half-maximal inhibitory concentration (IC_50_) values were calculated by linear regression analysis of the concentration–response curves.

### 2.8. Cell Culture

The immortalized human keratinocyte cell line (HaCaT) was kindly provided by Professor Dr. Suna Sabuncuoğlu (Department of Pharmaceutical Toxicology, Faculty of Pharmacy, Hacettepe University). The murine macrophage cell line (RAW 264.7) was used to evaluate the anti-inflammatory activity of the extract and sub-fractions. Both cell lines were cultured in DMEM supplemented with 10% FBS, 1% L-glutamine, and 0.5% penicillin–streptomycin and maintained at 37 °C in a humidified atmosphere containing 5% CO_2_ during experiments. All cell-culture reagents (DMEM, FBS, L-glutamine, penicillin–streptomycin) were obtained from Sigma-Aldrich (St. Louis, MO, USA), as detailed in Section 2.1.1.

### 2.9. Cell Viability Assay

Cell viability was evaluated using the MTT assay according to the method of Mosmann with minor modifications [28,29].

RAW 264.7 and HaCaT cell suspensions were prepared at densities of 1 × 10^6^ cells/mL and 1 × 10^5^ cells/mL, respectively, and 100 µL of the cell suspension was dispensed into each well of a 96-well plate. The plates were incubated at 37 °C for 24 h. The culture medium was then aspirated and replaced with 100 µL of extract or fraction solutions prepared in complete culture medium at the indicated concentrations, followed by incubation for an additional 24 h. Subsequently, 10 µL of MTT solution (5 mg/mL in PBS) was added to each well.

For RAW 264.7 cells, plates were incubated for 4–6 h following MTT addition; the medium was then aspirated and replaced with 100 µL of DMSO to dissolve the resulting formazan crystals, and absorbance was measured at 577 nm with a reference wavelength of 655 nm. For HaCaT cells, plates were incubated for 4 h following MTT addition; 100 µL of 10% SDS solution was then added to dissolve the formazan crystals, and plates were incubated for a further 20 h before absorbance was measured at 577 nm with a reference wavelength of 655 nm.

A control group containing only culture medium (without sample) was included under identical experimental conditions. Cell viability (%) was calculated according to the following equation:

(3) **% Cell viability = 100 − (100 × [(A_control_ − A_sample_)/A_control_])**

where A_control_ is the absorbance of the control group and A_sample_ is the absorbance of the sample-treated group.

All experiments were performed in three independent experiments, each consisting of at least three replicate wells.

### 2.10. Evaluation of Anti-Inflammatory Activity by NO Inhibition in LPS-Stimulated RAW 264.7 Cells

The anti-inflammatory effects of the extract and fractions were evaluated in LPS-stimulated RAW 264.7 macrophages by assessing their inhibitory effects on NO production.

RAW 264.7 cell suspensions were prepared at a density of 1 × 10^6^ cells/mL, and 100 µL aliquots were seeded into 96-well plates. Plates were incubated at 37 °C for 24 h. The culture medium was then aspirated and replaced with 100 µL of extract or fractions prepared in complete culture medium (50, 100, 200, and 400 µg/mL), together with 100 µL of LPS solution (2 µg/mL); plates were then incubated for a further 24 h. Following incubation, 100 µL of the supernatant from each well was transferred to a new 96-well plate, and 100 µL of Griess reagent [1% sulfanilamide, 0.1% *N*-(1-naphthyl)ethylenediamine dihydrochloride, and 2.5% phosphoric acid in distilled water] was added. Absorbance was measured at 577 nm [30,31]. The adherent cells remaining in the wells after supernatant collection were subsequently used for the MTT assay, performed as described in Section 2.9.

The NO-inhibitory activity was evaluated by comparing nitrite levels in sample-treated wells with those in untreated control wells [LPS (−)] and LPS-stimulated control wells [LPS (+)]. The LPS-stimulated group served as the stimulated control. No standard anti-inflammatory reference drug was included in this assay. Nitrite concentrations were determined by comparison with a calibration curve constructed using known concentrations of sodium nitrite (NaNO_2_) treated with Griess reagent under identical conditions [32]. All experiments were performed in three independent experiments, each consisting of at least three replicate wells.

### 2.11. Evaluation of Cytoprotective Activity Against H_2_O_2_-Induced Oxidative Stress

The cytoprotective activity against H_2_O_2_-induced oxidative stress was evaluated using the MTT assay. Minoxidil, a topical agent with well-characterized effects on keratinocyte and follicular cell metabolism, was used as a pharmacologically active reference comparator to contextualize the magnitude of the responses observed for the plant fractions, rather than as a mechanistically matched antioxidant or anti-inflammatory control.

HaCaT cells were suspended at a density of 1 × 10^5^ cells/mL, and 100 µL of the cell suspension was dispensed into each well of a 96-well plate. The plates were incubated at 37 °C for 24 h. The culture medium was then aspirated and replaced with 100 µL of extract or fraction solutions prepared in complete culture medium at concentrations of 50, 100, 200, and 400 µg/mL or minoxidil solutions at the indicated concentrations, simultaneously with 100 µL of H_2_O_2_ solution (61.2 µg/mL) prepared in complete culture medium. The plates were incubated for 24 h.

Following treatment, cell viability was determined using the MTT assay as described in Section 2.9.

The cytoprotective effect against H_2_O_2_-induced oxidative damage was determined by comparing the absorbance of cells treated with the samples (extract, sub-fractions or minoxidil) in the presence of H_2_O_2_ with that of untreated control cells [H_2_O_2_ (–)] and H_2_O_2_-treated cells [H_2_O_2_ (+)]. Cell viability (%) was calculated according to the following equation:

(4) **% Cell viability = 100 − (100 × [(A_control_ − A_sample_)/A_control_])**

where A_control_ is the absorbance of the control group (medium only, no sample) and A_sample_ is the absorbance of the sample-treated group.

All experiments were performed in three independent experiments, each consisting of at least three replicate wells.

### 2.12. Metabolomic Analysis

#### 2.12.1. Sample Preparation

Metabolomic analyses were performed using the HaCaT cell line. Cells were seeded into 24-well plates at a density of 1 × 10^5^ cells/mL (500 µL per well) and incubated for 24 h at 37 °C in a humidified atmosphere containing 5% CO_2_. The culture medium was then aspirated and replaced with 500 µL of extract or sub-fraction solutions (200 µg/mL) prepared in complete culture medium, together with 500 µL of H_2_O_2_ solution (61.2 µg/mL) prepared in complete culture medium, followed by incubation for an additional 24 h. Experimental groups included an untreated control [H_2_O_2_ (−)], an H_2_O_2_-induced oxidative stress group [H_2_O_2_ (+)], groups co-treated with H_2_O_2_ and each extract or fraction, groups treated with the extract or fractions alone (200 µg/mL, without H_2_O_2_) to evaluate metabolic alterations induced by the extract or fractions independent of oxidative stress, and a separate minoxidil-only group (100 µM), included for descriptive comparison rather than as an antioxidant or oxidative-stress positive control. Fraction and H_2_O_2_ exposure began simultaneously in the co-treatment groups; the design did not test treatment after oxidative injury had been established.

Following incubation, the culture medium was removed, and the cells were washed with phosphate-buffered saline to eliminate extracellular metabolites. Metabolic activity was rapidly quenched by briefly cooling the plates on liquid nitrogen for approximately 30 s. Methanol (500 µL) was then added to each well, and the cells were thoroughly scraped and transferred to microcentrifuge tubes. This extraction step was repeated once more. Cell counts were recorded for each biological replicate and used for subsequent normalization of the LC–MS peak intensities. Samples were stored at −80 °C until further processing.

#### 2.12.2. Sample Preparation for LC/MS Analysis

Samples were thawed at room temperature for 20 min, vortexed for 1 min, and centrifuged at 12,000 rpm for 25 min at 4 °C to precipitate proteins and obtain a clear supernatant. Following centrifugation, 0.5 mL of the supernatant was transferred to a new tube and dried. The dried samples were reconstituted in 0.2 mL of cold acetonitrile: water (1:1, *v*/*v*), vortexed for 1 min, and centrifuged again under the same conditions to remove residual particulates. Finally, 0.1 mL of the clarified supernatant was transferred to glass vials for LC/MS analysis. All experimental groups comprised three independent biological replicates, each analyzed in duplicate. Following normalization to the cell count, the paired analytical measurements were averaged within each biological replicate before statistical analysis. If only one injection yielded a detectable signal for a metabolite, that measurement represented the biological replicate; if neither did, the biological replicate was treated as missing.

#### 2.12.3. LC/MS Conditions

Metabolomic profiling was performed using a Q-TOF LC/MS system. Chromatographic separation was carried out at 35 °C using a reversed-phase column at a flow rate of 0.3 mL/min. The mobile phases consisted of water containing 0.1% formic acid (A) and acetonitrile containing 0.1% formic acid (B). Gradient elution was performed as follows: 95% A at 0 min, 65% A at 2 min, 5% A at 8 min, returning to 95% A at 10 min, and held at 95% A for 5 min for column re-equilibration. MS^1^ spectra were acquired over an *m*/*z* range of 75–1200, and sample injection order was randomized to minimize batch effects. Cellular metabolomic analyses were performed in negative electrospray ionization mode [ESI (−)] with the following parameters: capillary voltage, 3.5 kV; gas temperature, 325 °C; drying gas flow, 10 L/min; nebulizer pressure, 35 psi; fragmentor voltage, 175 V; skimmer voltage, 65 V. Continuous mass axis calibration was maintained using a reference mass solution, and blank samples were included in the analytical sequence to assess background contamination. A pooled quality-control sample was injected after every six sample injections to monitor analytical stability throughout the sequence.

#### 2.12.4. Data Processing and Statistical Analysis

Raw LC-MS data files were processed using MZmine 2.53 software. Raw data processing included mass detection, chromatogram building, peak deconvolution, isotope grouping, feature alignment, gap filling, and generation of the feature list. Mass detection was performed with an intensity threshold of 1000, and alignment was carried out with an *m*/*z* tolerance of 8 ppm and a retention time tolerance of 0.1 min. To minimize background signals originating from blank samples, only features with a sample-to-blank peak intensity ratio greater than 3 were retained for further analysis. Following pre-processing, peak intensities were normalized to the experimentally determined cell count of the corresponding biological sample. Paired analytical injections were averaged within each biological replicate after cell-count normalization.

Metabolite annotation followed Metabolomics Standards Initiative (MSI) recommendations [33]. Annotations matched to authentic standards in the in-house library by accurate mass and retention time were classified conservatively as MSI Level 2; accurate-mass matches to Human Metabolome Database (HMDB) or METLIN without in-house library matches were classified as MSI Level 3. MS/MS spectra were not acquired and no metabolite reached MSI Level 1. All names, especially those of lipid mediators assigned at Level 3, therefore denote putative annotations. PCA, PLS-DA, VIP estimation, heatmaps and the revised univariate statistical tests were computed in Python 3.13.16 with SciPy 1.17.0 and scikit-learn 1.8.0. PCA used log_2_ intensities from the 64 metabolites detected in all 21 biological samples, followed by autoscaling. Supplementary heatmaps show biological samples, metabolite-wise standardized and hierarchically clustered for descriptive visualization.

For the overall seven-group comparison, metabolites observed in at least two independent biological replicates per group were log_2_-transformed and analyzed with Welch one-way ANOVA; missing values were not imputed. Benjamini–Hochberg correction was applied to all metabolites eligible for this analysis, with q < 0.05 indicating an overall group difference. Eight prespecified pairwise comparisons used two-sided Welch t-tests on log_2_-transformed biological-replicate intensities for metabolites observed in at least two replicates in each compared group. Pairwise results satisfying unadjusted *p* < 0.05 and a fold change (FC) ≥ 1.5 or ≤0.67 are presented solely as exploratory candidates, without multiplicity-adjusted pairwise inference. FC is the ratio of untransformed group means. Four exploratory PLS-DA models were fitted using 64 complete-case metabolites and two prespecified components. Class-response R^2^ and cross-validated Q^2^ were assessed using three-fold stratified cross-validation, with autoscaling fitted on each training fold, and 199 class-label permutations. VIP scores describe contributions to the fitted supervised models and are not metabolite-level significance tests. The KEGG associations are a descriptive network and were not used to infer enriched or altered pathways.

### 2.13. Statistical Analysis

All experiments were performed in three independent experiments, and the results are presented as the mean ± standard error of the mean (SEM). Statistical analyses were performed using GraphPad Prism version 11 (GraphPad Software, San Diego, CA, USA). For the primary efficacy endpoints (NO-inhibitory activity and H_2_O_2_-induced cytoprotection), differences among groups were analyzed by one-way analysis of variance (ANOVA), followed by Dunnett’s multiple comparisons test, with the control group used as the reference. The DPPH and superoxide radical-scavenging results were evaluated descriptively to characterize concentration–response profiles. No inferential statistical analyses were performed for these datasets; therefore, statistical significance symbols were not included. Cell viability/cytotoxicity screening assays were evaluated descriptively, by comparing viability percentages relative to the untreated or LPS-stimulated control, rather than by inferential statistical testing, consistent with their role as a prerequisite screening step to establish non-cytotoxic concentration ranges rather than a hypothesis-testing endpoint. A *p* value < 0.05 was considered statistically significant.

## 3. Results

### 3.1. Total Phenolic and Flavonoid Contents

The total phenolic and flavonoid contents of the 80% ethanol extract and its *n*-hexane, ethyl acetate, *n*-butanol, and water fractions were determined.

Total phenolic content ranged from 12.27 to 51.84 mg GAE/g dry extract. The ethyl acetate fraction exhibited the highest total phenolic content (51.84 mg GAE/g dry extract), followed by the *n*-hexane (40.59 mg GAE/g dry extract), *n*-butanol (33.34 mg GAE/g dry extract), and crude ethanol extract (25.52 mg GAE/g dry extract), whereas the water fraction contained the lowest level (12.27 mg GAE/g dry extract) (Table 1).

A similar distribution was observed for total flavonoid content, which ranged from 9.92 to 176.56 mg QE/g dry extract. The ethyl acetate fraction showed the highest flavonoid content (176.56 mg QE/g dry extract), exceeding that of the *n*-hexane fraction (70.77 mg QE/g dry extract) by more than twofold. The water fraction exhibited the lowest flavonoid content (9.92 mg QE/g dry extract) (Table 1).

### 3.2. Antioxidant Activity

#### 3.2.1. DPPH Radical Scavenging Activity

**Figure 1:**
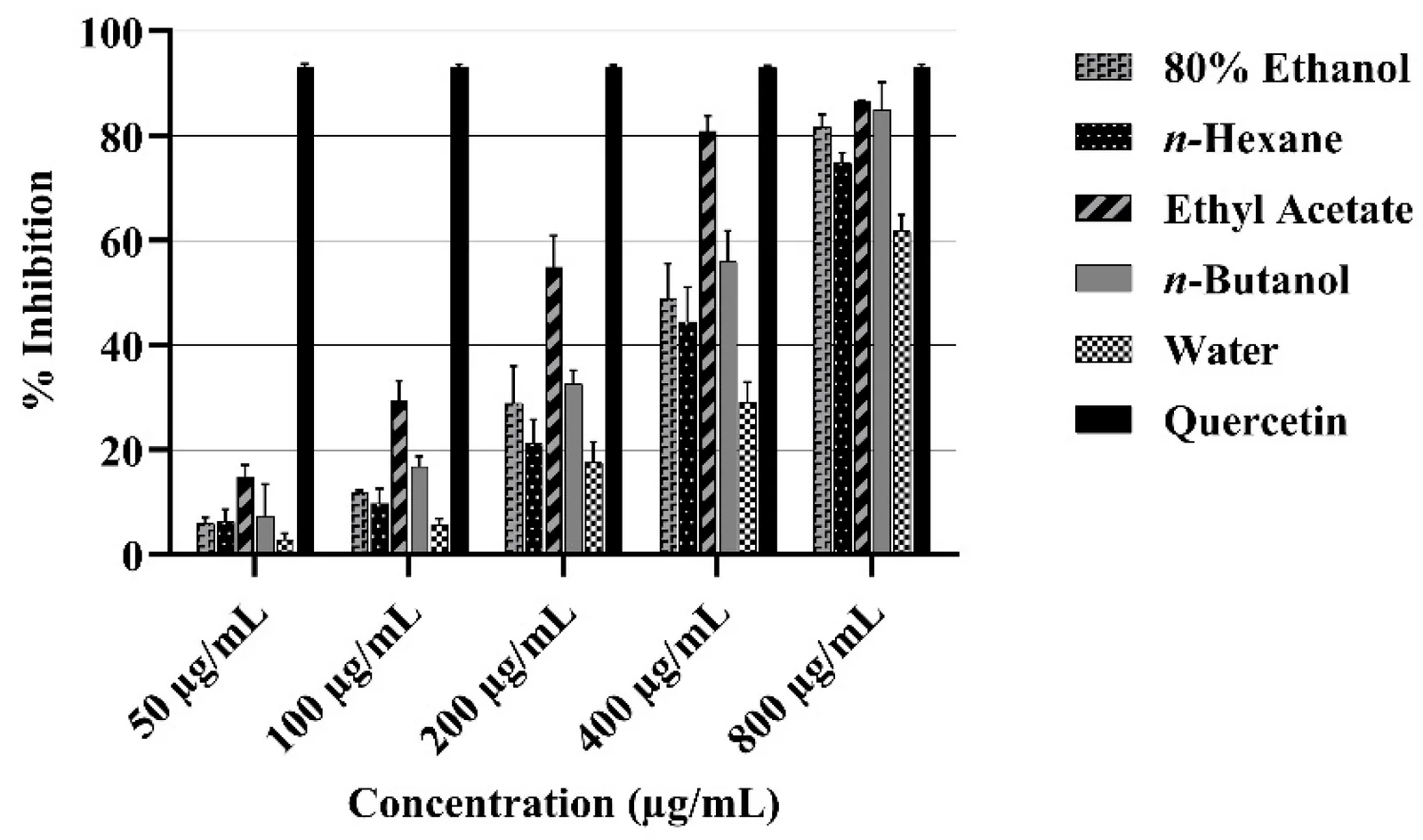
DPPH radical scavenging activity of the 80% ethanol extract, sub-fractions of *S. dichotoma* Ehrh. subsp. *dichotoma*, and quercetin.

The DPPH radical scavenging activity of the extract and fractions was evaluated at concentrations ranging from 50 to 800 µg/mL, using quercetin as the positive control. All samples exhibited concentration-dependent radical scavenging activity (Figure 1). Among the tested samples, the ethyl acetate fraction showed the highest activity (IC_50_ = 180.48 µg/mL), whereas the water fraction was the least active (IC_50_ = 655.57 µg/mL) (Table 2). At 800 µg/mL, inhibition reached 86.52% and 85.01% for the ethyl acetate and *n*-butanol fractions, respectively, while quercetin exhibited approximately 93% radical scavenging activity across the tested concentration range.

#### 3.2.2. SO Radical Scavenging Activity

**Figure 2:**
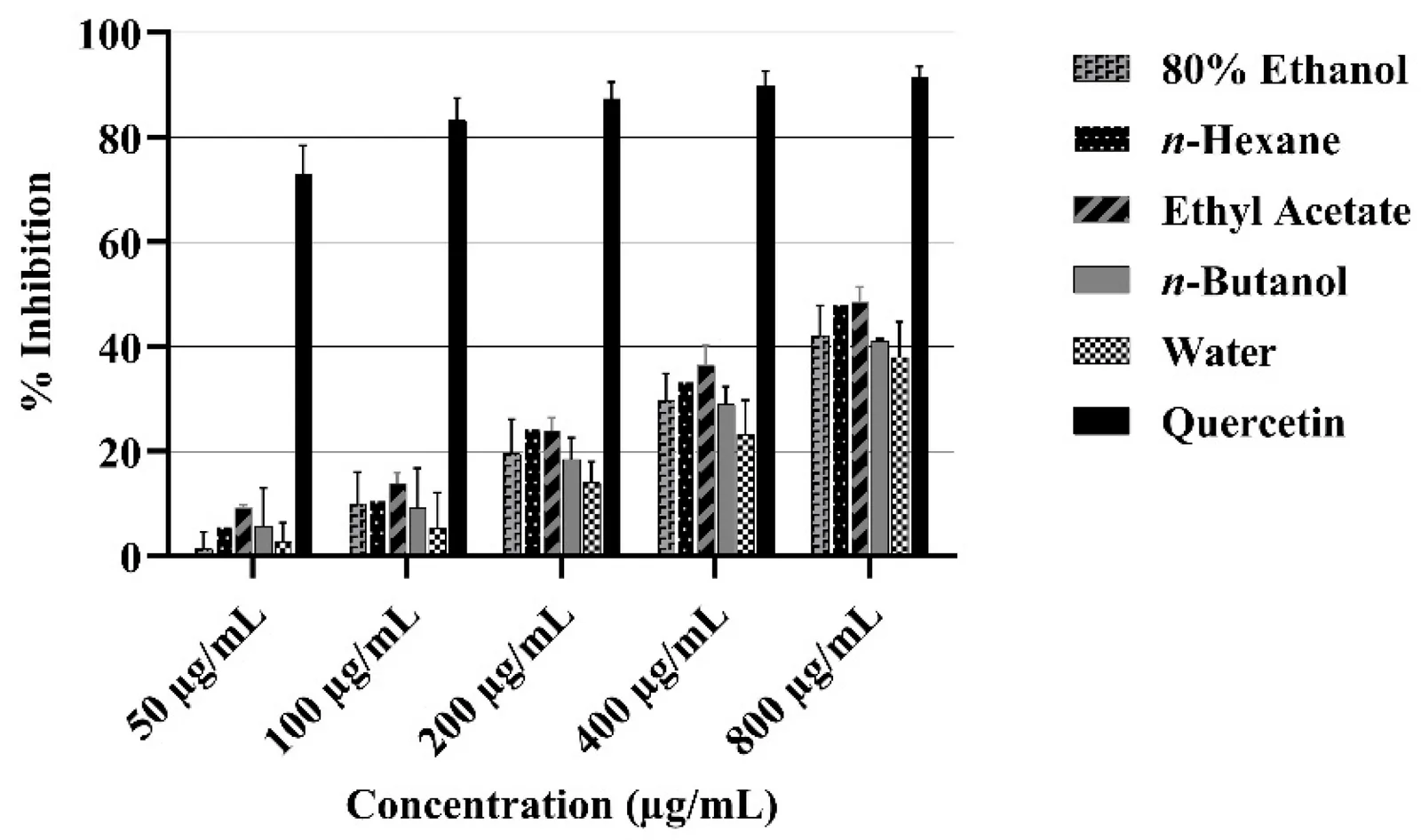
SO radical scavenging activity of the 80% ethanol extract, sub-fractions of *S. dichotoma* Ehrh. subsp. *dichotoma*, and quercetin.

The SO radical scavenging activity of the extract and fractions was evaluated at concentrations ranging from 50 to 800 µg/mL, using quercetin as the positive control. All samples exhibited concentration-dependent radical scavenging activity; however, none reached 50% inhibition within the tested concentration range, resulting in IC_50_ values > 800 µg/mL. At 800 µg/mL, scavenging activity ranged from 38.07% to 48.65%, with the ethyl acetate fraction showing the highest activity (48.65%), followed closely by the *n*-hexane fraction (48.19%) (Figure 2).

### 3.3. Characterization of the Isolated Compounds

**Figure 3:**
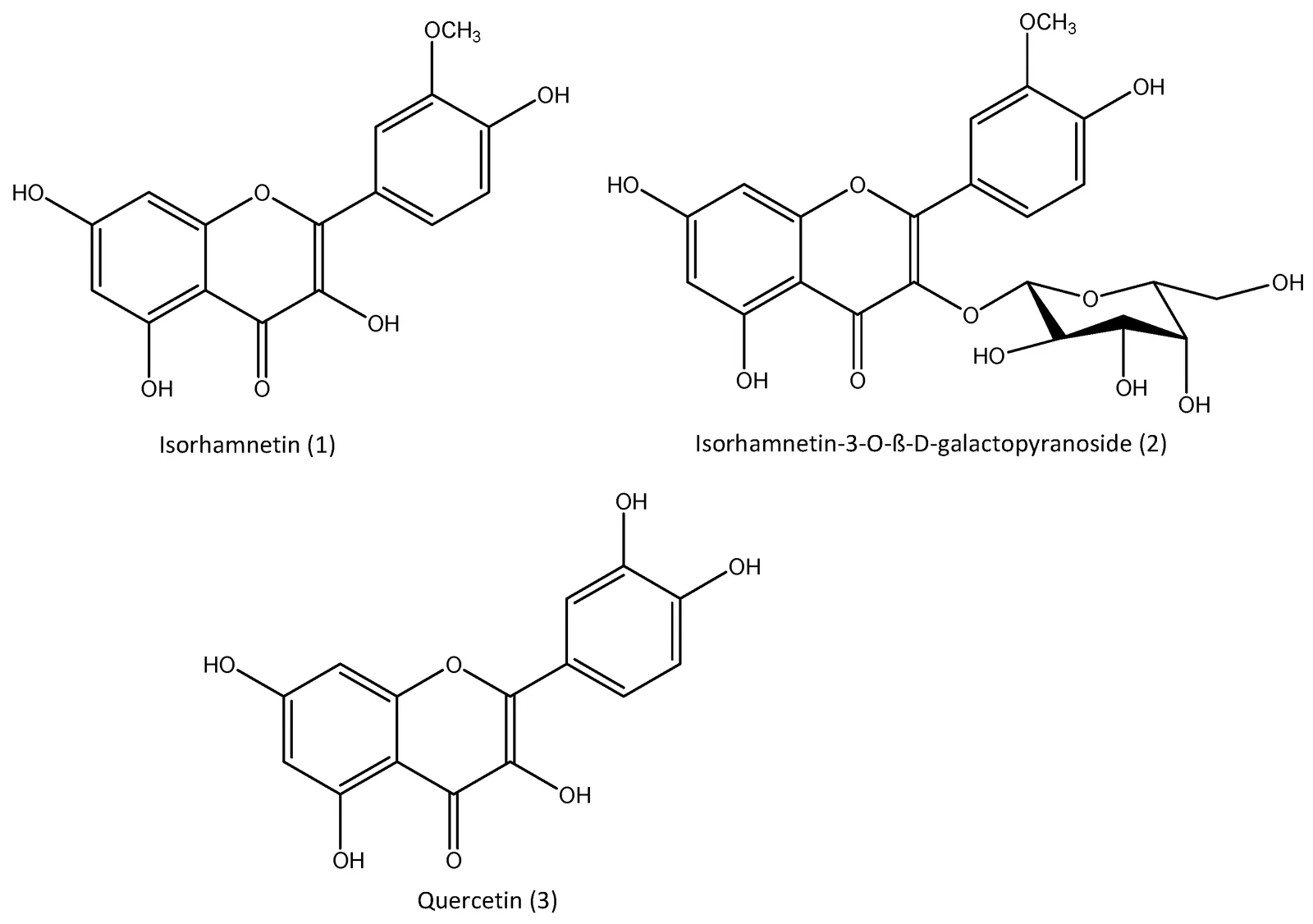
Chemical structures of the flavonoids isolated from the ethyl acetate fraction of *S. dichotoma* Ehrh. subsp. *dichotoma*: isorhamnetin (1), isorhamnetin-3-*O*-β-D-galactopyranoside (2), and quercetin (3).

Phytochemical investigation of the ethyl acetate fraction obtained from the aerial parts of *S. dichotoma* Ehrh. subsp. *dichotoma* resulted in the isolation of three flavonoids. Their structures were elucidated using 1D- and 2D-NMR and ESI-MS analyses and confirmed by comparison with published literature data [34,35,36]. The isolated compounds were identified as isorhamnetin (1), isorhamnetin-3-*O*-β-D-galactopyranoside (2), and quercetin (3) (Figure 3). To the best of our knowledge, this is the first report of the isolation of these compounds from *S. dichotoma* Ehrh. subsp. *dichotoma*.

### 3.4. Cell Viability

#### 3.4.1. Effect on RAW 264.7 Cell Viability

**Figure 4:**
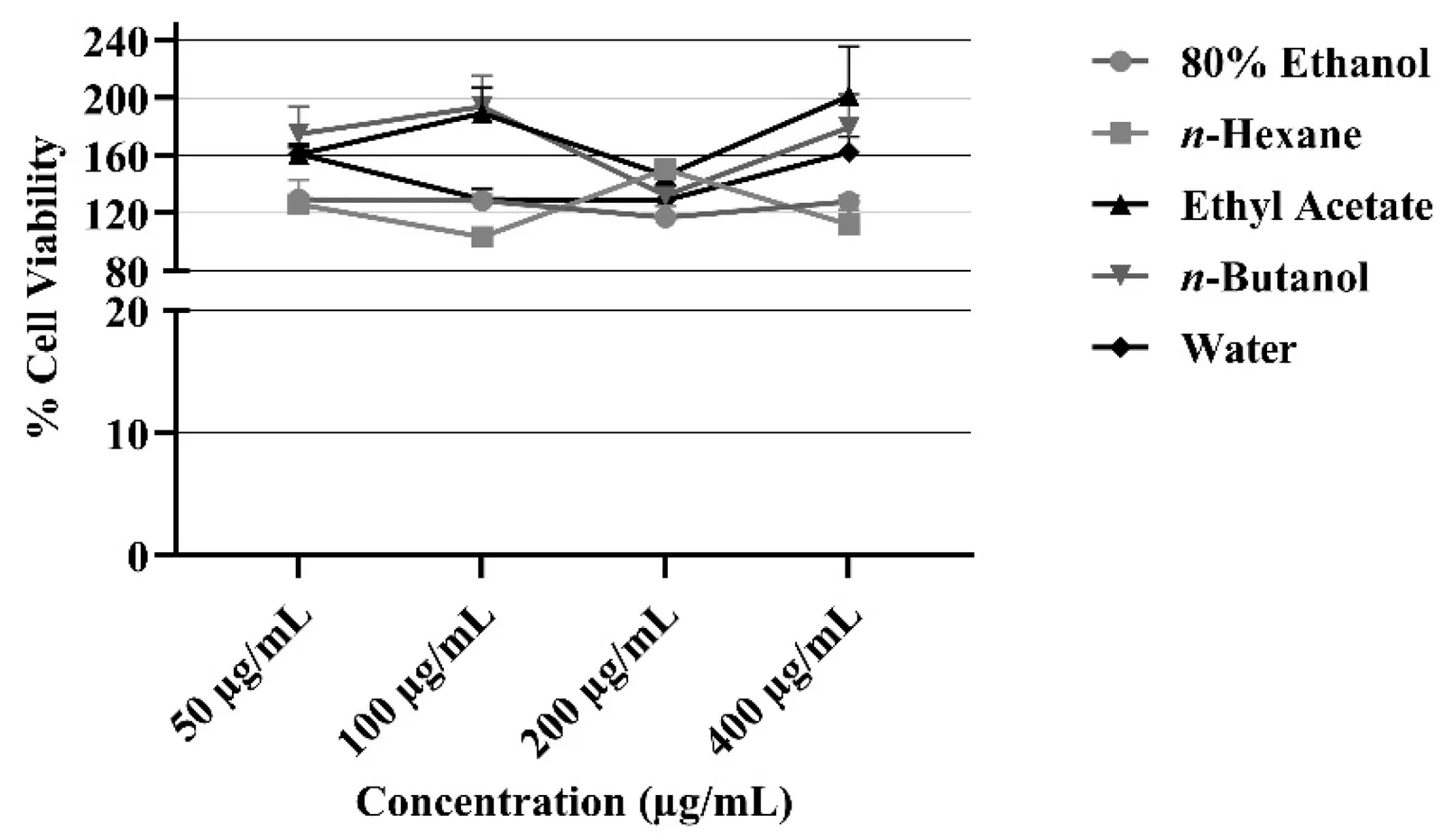
Effects of the 80% ethanol extract and sub-fractions of *S. dichotoma* Ehrh. subsp. *dichotoma* on RAW 264.7 cell viability.

The effects of the 80% ethanol extract and its fractions on RAW 264.7 cell viability were evaluated by MTT assay at concentrations of 50–400 µg/mL. None of the samples exhibited cytotoxicity within the tested concentration range, and MTT responses remained at or above those of the untreated control (Figure 4). Therefore, concentrations of 50–400 µg/mL were selected for subsequent anti-inflammatory assays in LPS-stimulated RAW 264.7 cells.

#### 3.4.2. Effect on HaCaT Cell Viability

**Figure 5:**
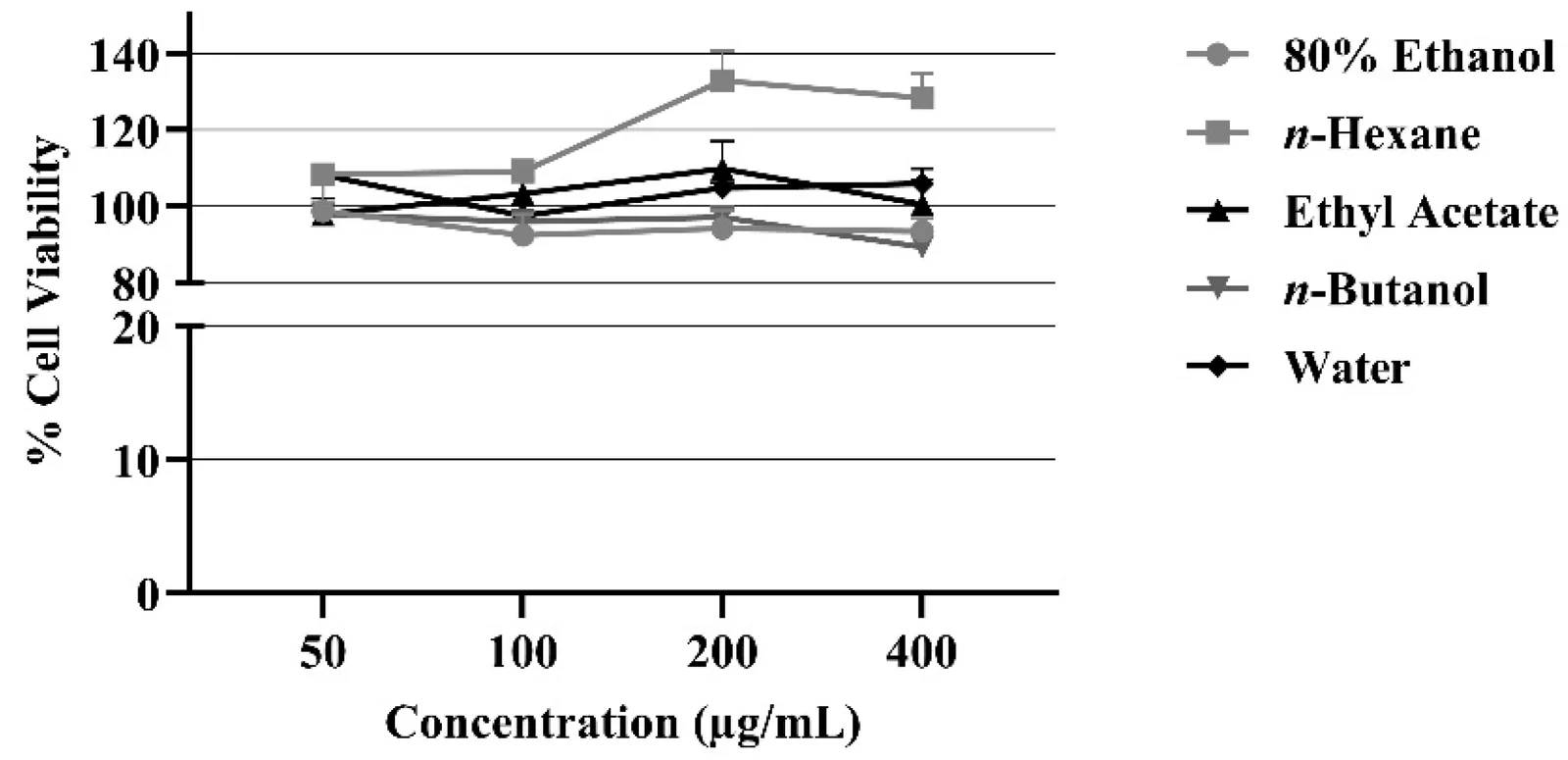
Effects of the 80% ethanol extract and sub-fractions of *S. dichotoma* Ehrh. subsp. *dichotoma* on HaCaT cell viability.

The effects of the 80% ethanol extract and its fractions on HaCaT cell viability were evaluated to determine non-cytotoxic concentrations for subsequent cytoprotective assays. Minoxidil, used as the reference comparator, was tested at concentrations ranging from 3.9 to 500 µM. None of the samples or minoxidil exhibited cytotoxicity within the tested concentration ranges, with cell viability remaining above 89.29% in all groups (Figure 5). Among the fractions, the *n*-hexane fraction produced the highest MTT response, reaching 132.92% at 200 µg/mL, while the remaining extract and fractions generally maintained cell viability close to or above that of the untreated control. These concentrations were therefore considered suitable for subsequent evaluation of cytoprotective activity against H_2_O_2_-induced oxidative stress.

### 3.5. Anti-Inflammatory Activity in LPS-Stimulated RAW 264.7 Cells

**Figure 6:**
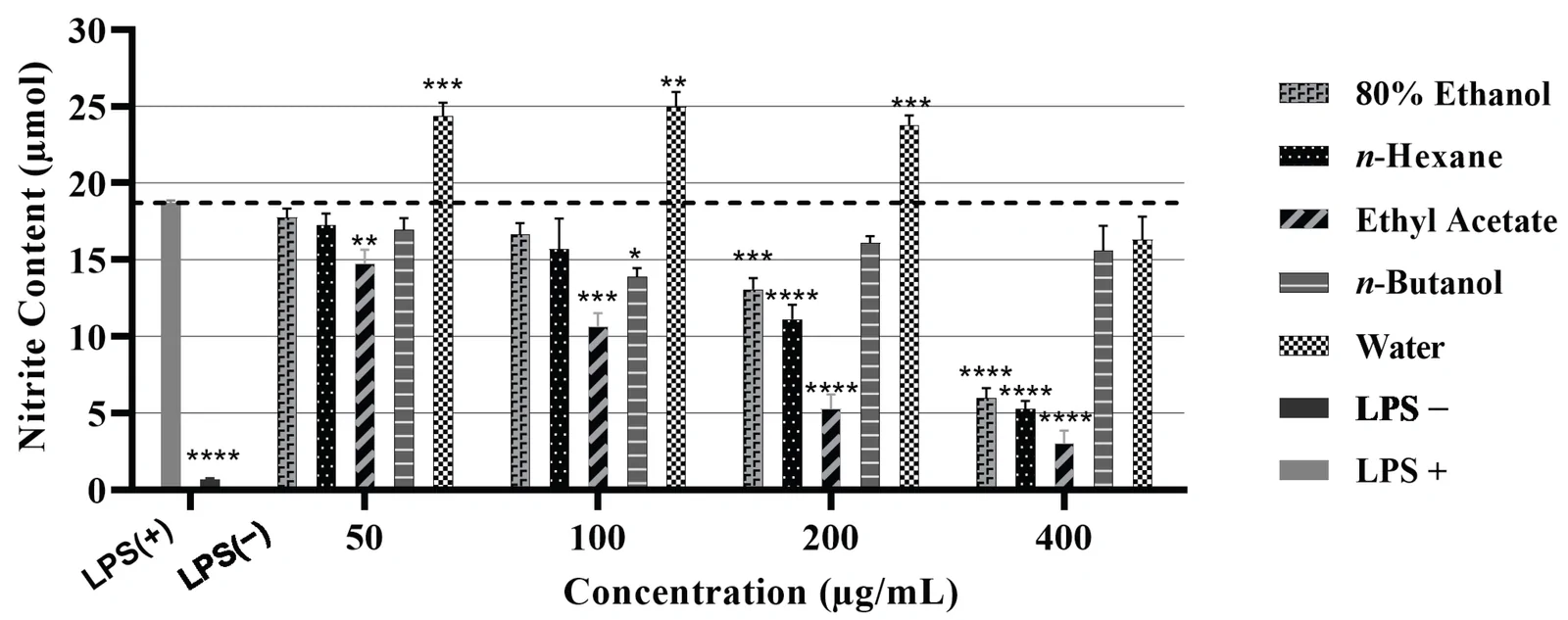
Inhibitory effects of the 80% ethanol extract and sub-fractions of *S. dichotoma* Ehrh. subsp. *dichotoma* on NO production in LPS-stimulated RAW 264.7 macrophages. LPS (+) stimulated control; LPS (−), negative control. * *p* < 0.05, ** *p* < 0.01, *** *p* < 0.001, **** *p* < 0.0001 vs. LPS (+); comparisons without a symbol were not statistically significant (*p* > 0.05).

The effects of the 80% ethanol extract and its fractions on NO production were evaluated in LPS (2 µg/mL)-stimulated RAW 264.7 macrophages at non-cytotoxic concentrations of 50–400 µg/mL using the Griess assay. LPS stimulation increased nitrite levels from 0.65 µM in unstimulated cells [LPS (−)] to 18.71 µM [LPS (+)]. The ethyl acetate fraction showed the strongest concentration-dependent effect, reducing nitrite levels from 14.75 µM at 50 µg/mL to 3.00 µM at 400 µg/mL (Figure 6). The 80% ethanol extract and *n*-hexane fraction also reduced nitrite levels in a concentration-dependent manner, reaching 5.95 and 5.30 µM at 400 µg/mL, respectively. In contrast, the *n*-butanol fraction showed no clear concentration-dependent effect, while the water fraction increased nitrite levels at 50–200 µg/mL and produced only a modest reduction at 400 µg/mL (reduced nitrite levels to 16.31 µM).

**Figure 7:**
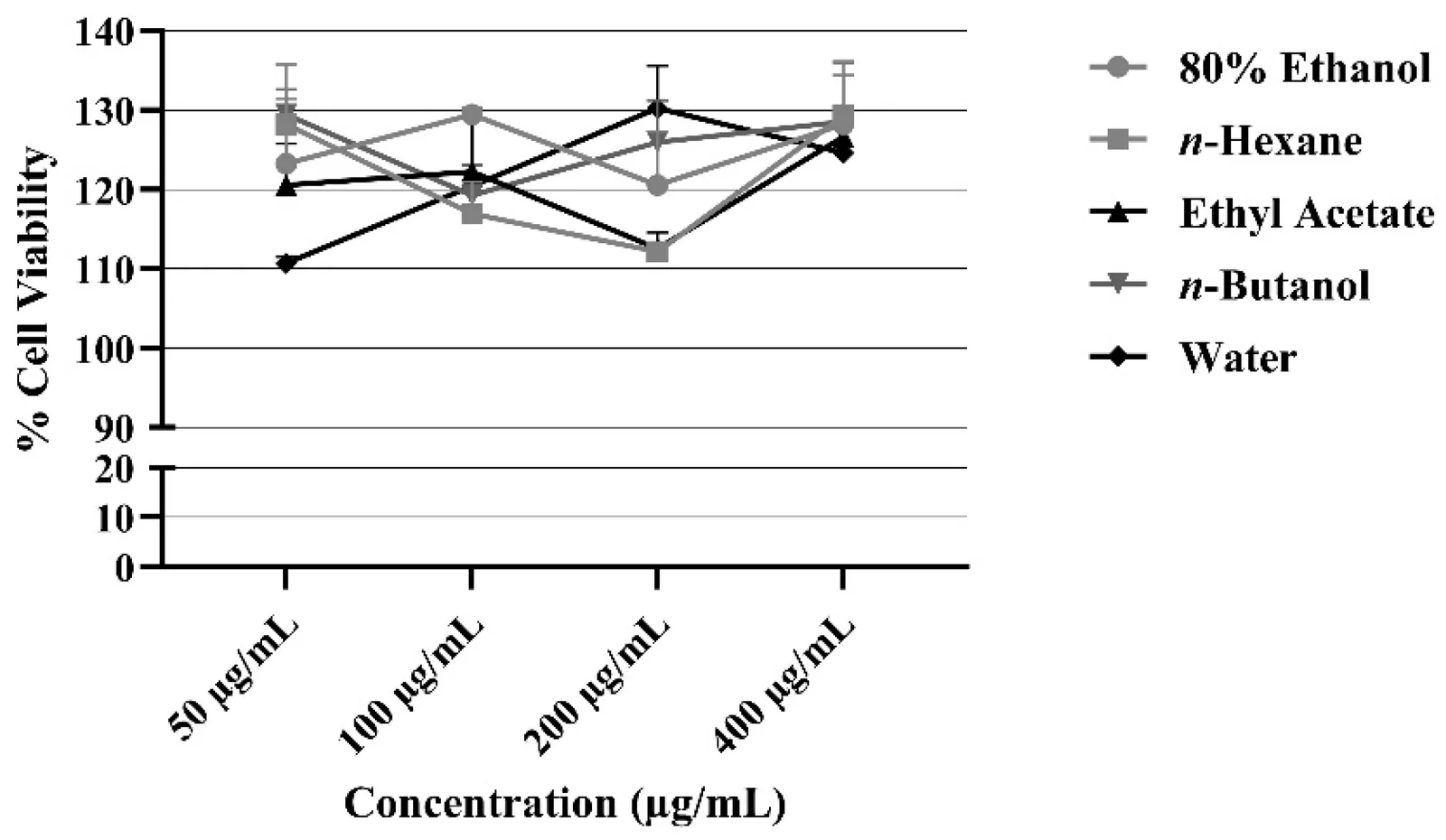
Effects of the 80% ethanol extract and sub-fractions of *S. dichotoma* Ehrh. subsp. *dichotoma* on the viability of LPS-stimulated RAW 264.7 macrophages.

To confirm that the observed reductions in NO production were not attributable to cytotoxicity, cell viability was evaluated in LPS-stimulated RAW 264.7 cells under the same experimental conditions (Figure 7). None of the treatments reduced cell viability following LPS stimulation, with viability remaining at or above that of the LPS-treated control. These findings indicate that the reductions in NO production observed for the active samples were not associated with cytotoxicity.

### 3.6. Cytoprotective Effect Against H_2_O_2_-Induced Oxidative Stress in HaCaT Cells

**Figure 8:**
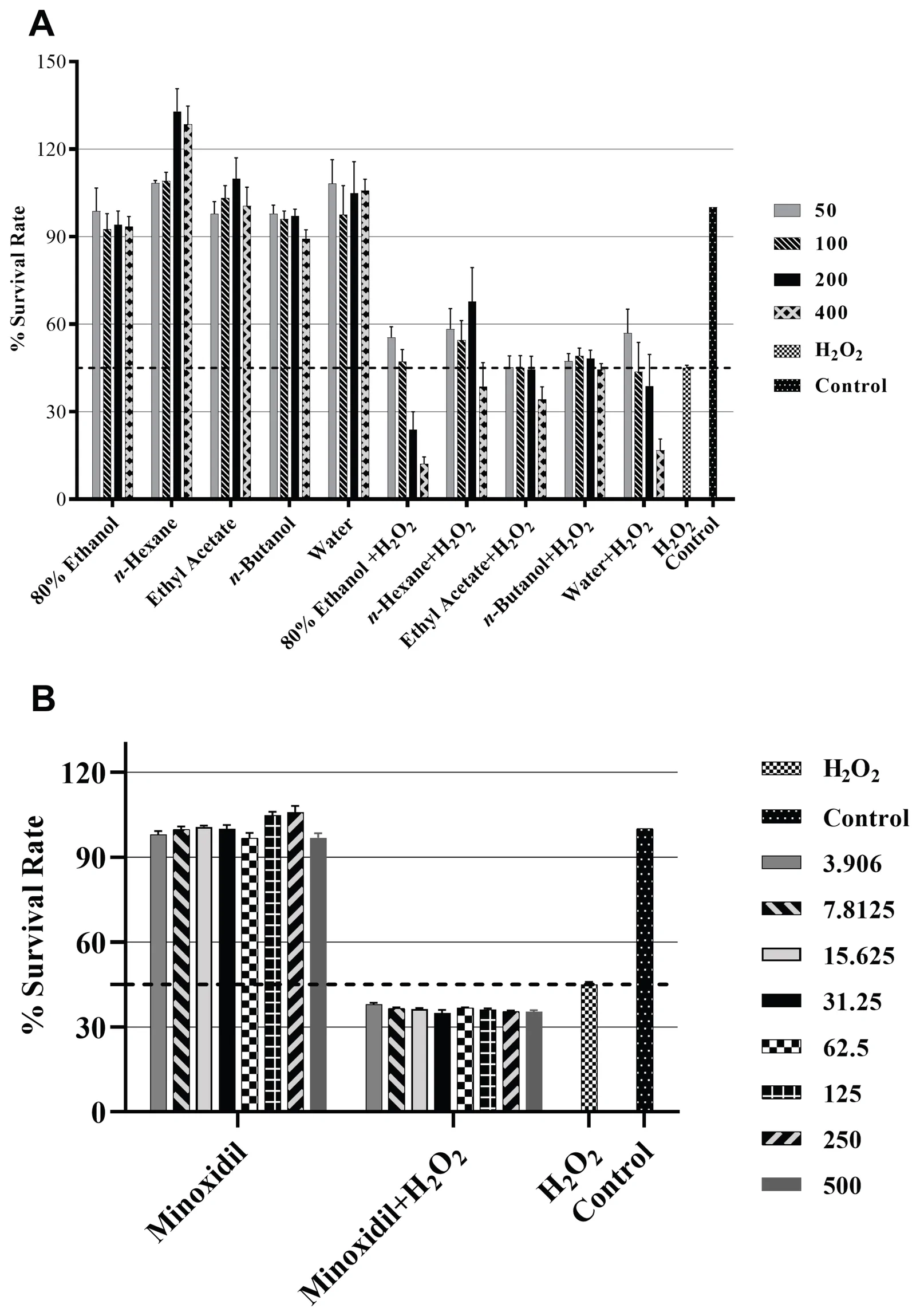
Effects of the 80% ethanol extract, sub-fractions, and minoxidil on H_2_O_2_-induced oxidative damage in HaCaT cells. (**A**) 80% ethanol extract and sub-fractions (*n*-hexane, ethyl acetate, *n*-butanol, aqueous). (**B**) Minoxidil. No treatment produced a statistically significant protective effect relative to the H_2_O_2_-only group (one-way ANOVA with Dunnett’s post hoc test, *p* > 0.05 for all comparisons); accordingly, no significance symbols are shown.

The effects of the 80% ethanol extract and its fractions on H_2_O_2_-induced oxidative damage were evaluated in HaCaT cells at non-cytotoxic concentrations, with minoxidil included as a reference comparator. H_2_O_2_ treatment reduced cell viability to 45.02% relative to the untreated control (100%). None of the extract, fractions, or minoxidil produced a statistically significant cytoprotective effect under the tested conditions (Figure 8A,B). The *n*-hexane fraction showed the highest numerical viability (67.77%) at 200 µg/mL, although viability decreased to 38.67% at 400 µg/mL. In contrast, the 80% ethanol extract and water fraction markedly reduced cell viability at higher concentrations, reaching 12.15% and 16.82%, respectively, at 400 µg/mL. The ethyl acetate and *n*-butanol fractions produced viability values comparable to those of the H_2_O_2_-treated control across the tested concentrations. Minoxidil similarly showed no significant protective effect.

### 3.7. Metabolomic Analysis

#### 3.7.1. Metabolite Profiling and Exploratory Visualization

LC/MS-based untargeted metabolomic analysis was performed to characterize metabolic alterations associated with H_2_O_2_-induced oxidative stress and the effects of the *n*-hexane and ethyl acetate (EtOAc) fractions in HaCaT cells. The ethyl acetate fraction had the strongest DPPH response, whereas the *n*-hexane fraction was selected as a chemically contrasting, less polar fraction with measurable DPPH and NO-assay responses. The 200 µg/mL concentration was selected for the *n*-hexane fraction in the metabolomic experiments because it corresponded to the highest numerical viability observed for this fraction during H_2_O_2_ co-treatment (67.77%), although statistically significant protection was not demonstrated. At 400 µg/mL, viability during co-treatment was lower (38.67%); therefore, this higher concentration was not used for metabolomic profiling. All fractions had superoxide-scavenging IC_50_ values above 800 µg/mL; this assay did not provide evidence of potent activity or justify selecting *n*-hexane. Minoxidil was included as a separate treatment-only, descriptive comparator, not as a positive control for H_2_O_2_ protection.

After quality-control filtering, 99 metabolites remained putatively annotated (51 MSI Level 2 and 48 MSI Level 3; Table S1). Seventy-five metabolites were observed in at least two independent biological replicates in every group and were eligible for the overall seven-group analysis; 38 showed overall differences after Benjamini–Hochberg (BH) correction (q < 0.05; Table S2). Throughout Section 3.7.2, Section 3.7.3, Section 3.7.4 and Section 3.7.5, pairwise candidates were selected using unadjusted *p* < 0.05 and FC ≥ 1.5 or ≤0.67. These comparisons are exploratory, and higher or lower intensity refers to the direction of the group-mean difference.

Four prespecified PLS-DA models considered (i) Control, H_2_O_2_, *n*-Hexane Fr., and *n*-Hexane Fr. + H_2_O_2_; (ii) Control, H_2_O_2_, EtOAc Fr., and EtOAc Fr. + H_2_O_2_; (iii) Control, *n*-Hexane Fr., EtOAc Fr., and Minoxidil; and (iv) all seven experimental groups. These supervised models and their VIP rankings were used for exploratory visualization, with cross-validation and label permutations summarized in Section 3.7.6.

**Figure 9:**
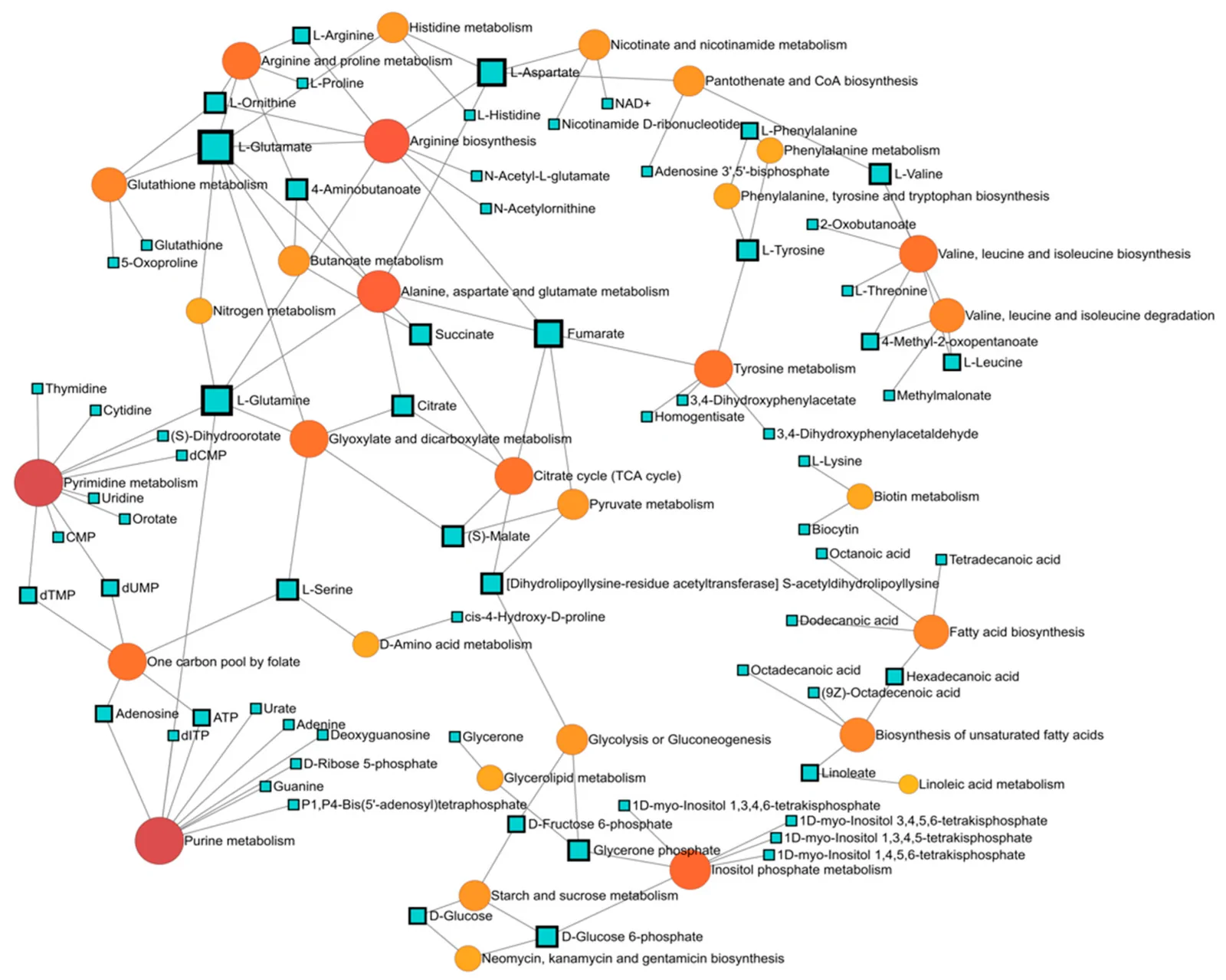
Descriptive KEGG metabolite–pathway network of the 99 putatively annotated metabolites. Connections represent database associations and do not indicate statistically significant pathway enrichment or changes in pathway activity.

Figure 9 maps KEGG database connections for the complete panel of 99 putative annotations.

**Figure 10:**
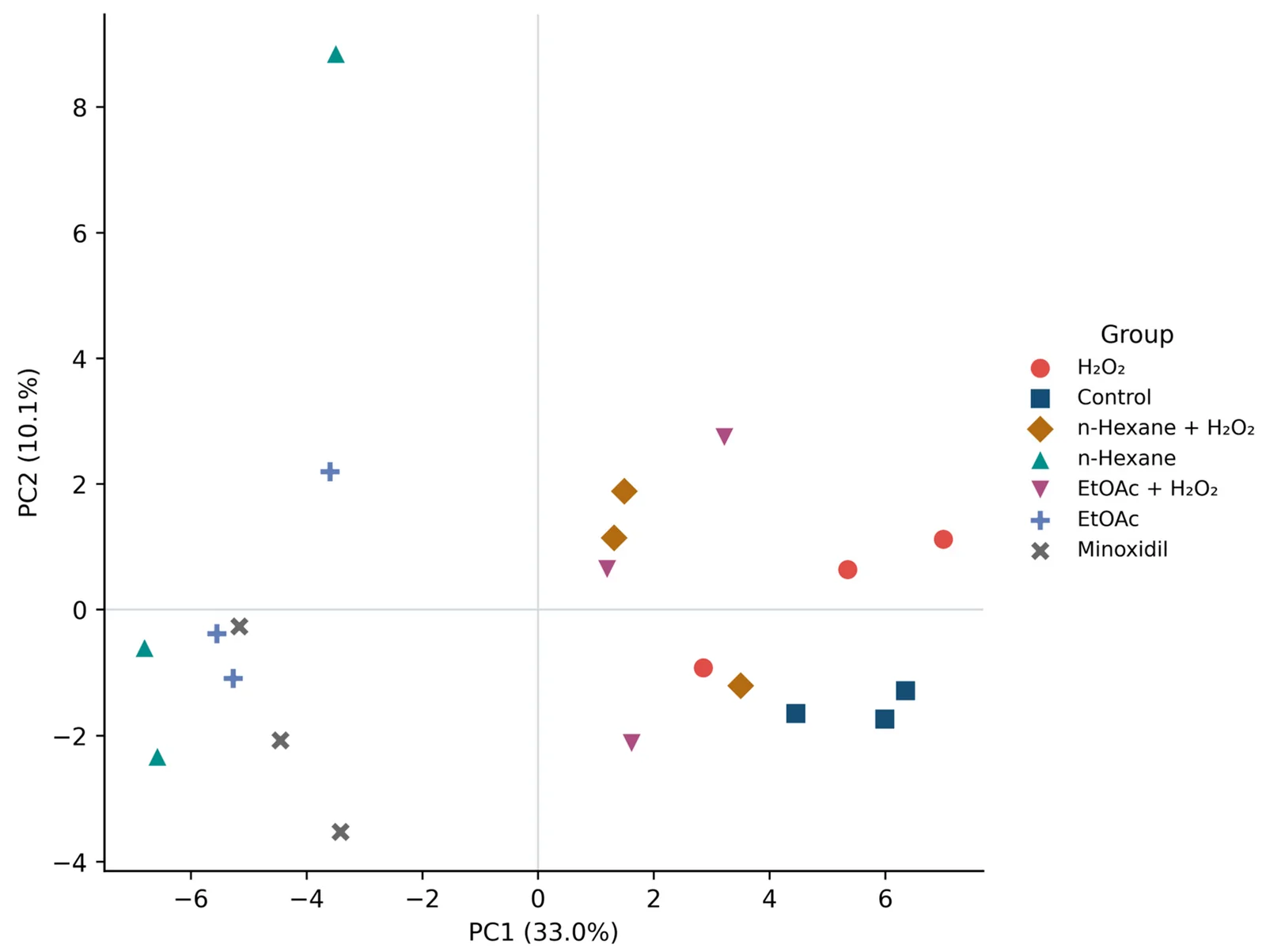
PCA score plot of 21 independent biological samples (three per group), based on 64 complete-case metabolites after averaging paired analytical injections, log_2_ transformation and autoscaling. PC1 and PC2 explain 33.0% and 10.1% of the variance, respectively. Each point represents one biological sample; the plot does not encode metabolite-level statistical significance.

PCA of the 21 independent biological samples and 64 complete-case metabolites explained 33.0% and 10.1% of the variance on principal component 1 (PC1) and PC2, respectively (Figure 10). The scores illustrate the distribution of individual biological samples; no test of group separation was performed.

#### 3.7.2. Metabolic Response to H_2_O_2_-Induced Oxidative Stress

In the H_2_O_2_ versus control comparison, nine metabolites met the exploratory criteria (seven lower and two higher mean intensities; Table S3). ATP and fructose 6-phosphate showed lower mean intensities, whereas adenine showed a higher mean intensity.

#### 3.7.3. Metabolomic Profile of the *n*-Hexane Fraction

Relative to control, 25 metabolites met the exploratory criteria following *n*-hexane fraction treatment alone (19 lower and six higher mean intensities; Table S4). Citrate, γ-aminobutyric acid, and a feature putatively annotated as S-hydroxymethylglutathione were among the lower-intensity candidates.

The two-component *n*-hexane four-group PLS-DA model yielded 34 metabolites with VIP > 1 (Table S5). The biological-replicate heatmap illustrates relative intensities for this four-group set (Figure S1), and model validation is reported in Section 3.7.6.

Relative to H_2_O_2_ alone, eight metabolites met the exploratory criteria after *n*-hexane + H_2_O_2_ co-treatment (six lower and two higher mean intensities; Table S6).

#### 3.7.4. Metabolomic Profile of the Ethyl Acetate Fraction

Relative to control, 28 metabolites met the exploratory criteria after EtOAc fraction treatment alone (24 lower and four higher mean intensities; Table S7). ATP and NAD showed lower mean intensities, whereas a feature putatively annotated as linoleic acid showed a higher mean intensity.

The two-component EtOAc four-group PLS-DA model yielded 32 metabolites with VIP > 1 (Table S8). Relative intensities at the biological-replicate level are displayed in the corresponding heatmap (Figure S2), and model validation is summarized in Section 3.7.6.

Relative to H_2_O_2_ alone, 16 metabolites met the exploratory criteria after EtOAc + H_2_O_2_ co-treatment (13 lower and three higher mean intensities; Table S9). The feature putatively annotated as 6-ketoprostaglandin E1 showed higher mean intensity.

#### 3.7.5. Comparative Metabolomic Profile of Minoxidil

Relative to control, 35 metabolites met the exploratory criteria after minoxidil treatment alone (31 lower and four higher mean intensities; Table S10).

The minoxidil comparative four-group PLS-DA model yielded 29 metabolites with VIP > 1 (Table S11). Figure S3 displays the associated biological-replicate intensity heatmap, and model validation is reported in Section 3.7.6.

Eight metabolites met the exploratory criteria for minoxidil versus *n*-hexane (all showing higher mean intensity in the minoxidil group; Table S12). Fourteen met the criteria for minoxidil versus EtOAc (five lower and nine higher in the minoxidil group; Table S13).

#### 3.7.6. Integrated Analysis Across All Experimental Groups

**Figure 11:**
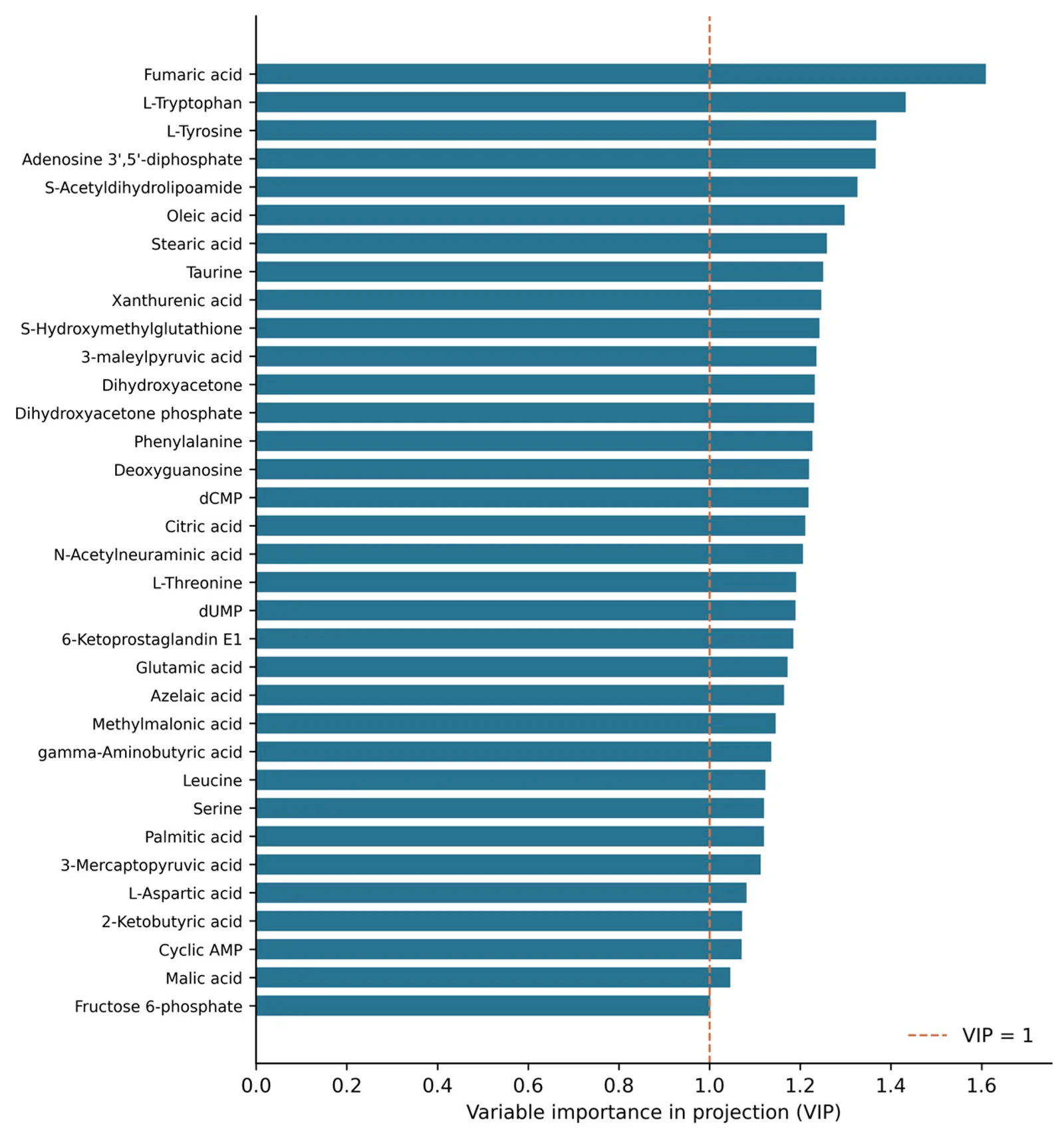
VIP scores from the two-component PLS-DA model of all seven groups, fitted to 21 independent biological samples and 64 complete-case metabolites. Thirty-four metabolites had VIP > 1. VIP scores rank variable contributions and are not metabolite-level hypothesis tests.

PLS-DA was refitted using independent biological samples and 64 complete-case metabolites. The *n*-hexane, EtOAc, and minoxidil four-group models had class-response R^2^/Q^2^ of 0.614/0.257, 0.637/0.387, and 0.613/0.367, respectively, with permutation *p* values of 0.005, 0.005, and 0.010 from 199 label permutations. The integrated seven-group model had R^2^ = 0.309, Q^2^ = 0.180, and permutation *p* = 0.005. The three biological replicates per group and modest integrated Q^2^ warrant cautious, exploratory interpretation of the supervised models. The integrated model yielded 34 metabolites with VIP > 1 (Figure 11); Figure S4 shows the descriptive biological-replicate heatmap.

Of 99 putatively annotated metabolites, 75 met the minimum biological-replicate detection criterion for the overall seven-group Welch ANOVA, and 38 showed an overall BH-adjusted difference (q < 0.05; Table S2). This group-level test does not identify which specific pairs of groups differ.

No pathway over-representation analysis is reported for the biological-replicate dataset. Figure 9 is a descriptive map of KEGG associations and does not represent statistically significant enrichment.

## 4. Discussion

*Silene* L. is a chemically diverse genus of the Caryophyllaceae, characterized by flavonoids, phytoecdysteroids, triterpene saponins, and terpenoids and associated with a broad range of reported biological activities, antioxidant, anti-inflammatory, and hair/scalp-relevant effects [7]. Oxidative stress and inflammation are also closely associated with hair follicle biology and have been implicated in disruption of the hair growth cycle [37,38].

*S. dichotoma* Ehrh. subsp. *dichotoma*, like several other *Silene* species, has traditionally been consumed as food [7]. Despite its traditional use and the broader phytochemical and pharmacological interest in the genus, *S. dichotoma* remains comparatively understudied. Previous investigations have largely focused on chromatographic characterization, while isolation studies and comprehensive cell-based investigations have remained limited [9,39]. To our knowledge, the present study is the first to integrate phytochemical characterization, compound isolation, antioxidant and cell-based biological evaluation, and metabolomic profiling of this taxon, providing a broader assessment of its biological potential.

Fractionation markedly enriched the phenolic and flavonoid content of the ethyl acetate fraction, which contained 51.84 mg GAE/g and 176.56 mg QE/g, respectively, and showed the strongest DPPH scavenging activity, with an IC_50_ of 180.48 µg/mL and 86.52% inhibition at 800 µg/mL. Superoxide radical scavenging was weaker, with IC_50_ values above 800 µg/mL for all samples. Previous analysis of a methanolic *S. dichotoma* extract reported total phenolic and flavonoid contents of 28.8 mg GAE/g and 47 mg rutin equivalents/g, respectively [9]. In a comparative study of six *Silene* species, the methanol extract of *S. dichotoma* produced 96 ± 3 mg Trolox equivalent (TE)/g in the DPPH assay, 134 ± 4 mg TE/g in the 2,2′-azino-bis(3-ethylbenzothiazoline-6-sulfonic acid) (ABTS) assay, 154 ± 1 mg TE/g in the cupric ion reducing antioxidant capacity (CUPRAC) assay, and 102 ± 1 mg TE/g in the ferric reducing antioxidant power (FRAP) assay; it also showed metal-chelating activity of 13 ± 1 mg ethylenediaminetetraacetic acid equivalent (EDTAE)/g and a phosphomolybdenum value of 1.1 ± 0.1 mmol TE/g [9]. *S. dichotoma* ranked highest for most of these parameters, providing context for the DPPH result but not changing the weak superoxide-scavenging response observed here (IC_50_ > 800 µg/mL for all samples).

Guided by these results, the ethyl acetate fraction was selected for phytochemical investigation. Quercetin, isorhamnetin, and isorhamnetin-3-O-β-D-galactopyranoside were isolated and characterized by spectroscopic methods [34,35,36]. To our knowledge, this is the first reported isolation of these compounds from *S. dichotoma* Ehrh. subsp. *dichotoma*. Their occurrence is consistent with the previously reported flavonoid profile of the species, including quercetin, rhamnetin, hyperoside, rutin, and isovitexin-type glycosides [9,39]. The previously reported antioxidant and anti-inflammatory activities of quercetin, isorhamnetin, and related derivatives [40,41,42,43,44,45,46,47] are consistent with the antioxidant and anti-inflammatory activities observed for the extract and fractions in the present study.

Traditional food use [7] reflects a history of oral consumption of this species and is presented here as ethnobotanical background. HaCaT cells are skin-derived keratinocytes and do not model intestinal exposure; therefore, the present cell-based findings do not establish oral safety or pharmacological effects following oral consumption. Cytotoxicity assessment in RAW 264.7 and HaCaT cells was performed to support the interpretation of subsequent cell-based biological effects under the experimental conditions used.

The absence of cytotoxicity in RAW 264.7 macrophages allowed the anti-inflammatory effects to be assessed across the tested concentration range. None of the samples reduced viability at 50–400 µg/mL; instead, viability values exceeded those of the control. This result differs from reports for other *Silene* species. The IC_50_ values of 50% methanol extracts of *S. succulenta* and *S. villosa* in RAW 264.7 cells were below 100 µg/mL, whereas that of *S. nocturna* was 100–150 µg/mL [48]. A methanol extract of *S. salsuginea* maintained viability at lower concentrations but caused a marked reduction above 31.25 µg/mL [49]. The high MTT values produced particularly by the ethyl acetate (145.97–201.07%) and *n*-butanol (132.12–193.86%) fractions may reflect increased mitochondrial metabolic activity, or proliferation of phenolic compounds, flavonoids, and phytoecdysteroids [11,28,50]. Because MTT reduction measures mitochondrial dehydrogenase activity, these possibilities cannot be separated by the MTT response alone.

Viability also remained above 100% when the extract and fractions were applied to LPS-stimulated RAW 264.7 macrophages. In activated macrophages, however, increased inducible NO synthase activity can accelerate enzymatic reduction in MTT and increase formazan formation, leading to an overestimation of viability [51]. Thus, the elevated values in the LPS-stimulated groups may include a method-related component in addition to genuine metabolic or proliferative effects. The ethyl acetate and *n*-butanol fractions, which produced the highest values in unstimulated cells, showed lower values after LPS stimulation (112.57–126.65% and 119.25–129.43%, respectively). Nevertheless, all treatments remained within a non-cytotoxic range, supporting the use of these concentrations in the NO assay.

In this study, anti-inflammatory potential was investigated using nitrite accumulation in LPS-stimulated RAW 264.7 cells as the experimental endpoint. No standard anti-inflammatory reference drug was included, which limits comparison with an established pharmacological benchmark. The observed reductions in nitrite accumulation demonstrate NO-inhibitory activity under the experimental conditions used and suggest potential anti-inflammatory relevance. Further assessment of additional inflammatory endpoints is required to establish broader anti-inflammatory effects.

LPS increased NO from 0.65 to 18.71 µM, whereas the ethyl acetate fraction produced the strongest inhibition (IC_50_ = 118.34 µg/mL), reducing NO to 3.00 µM at 400 µg/mL. The *n*-hexane fraction and 80% ethanol extract were less active (IC_50_ = 249.46 and 294.75 µg/mL), while the *n*-butanol and water fractions showed limited inhibition.

The NO results are consistent with anti-inflammatory findings reported for other *Silene* species, although the effective concentrations differ. At 10 µg/mL, a methanol extract of *S. succulenta* inhibited NO by 60.5%, while *S. nocturna* produced 30.8% inhibition and *S. villosa* showed a lower response; similar ranking was reported for cyclooxygenase-2 (COX-2) and TNF-α inhibition [48]. In a separate study, *S. succulenta* methanol extract reduced LPS-induced nitrite by 85% at 20 µg/mL, although 29% cytotoxicity was observed at that concentration [52]. *S. salsuginea* methanol extract reduced NO by 74.9% at a non-cytotoxic concentration of 15.6 µg/mL [49], and the ethyl acetate fraction of *S. vulgaris* inhibited COX-2 with an IC_50_ of 2.07 µg/mL [53]. In contrast, ethanol extracts of *S. sendtneri* and *S. roemeri* increased C-C motif chemokine ligand 18 (CCL-18), TNF-α, interleukin (IL)-1β, IL-4, and IL-6 production, indicating macrophage activation and an immunomodulatory profile [54]. Anti-inflammatory activity has also been demonstrated in vivo for *S. villosa* in carrageenan-induced paw edema and for the butanol fraction of *S. claviformis* in formalin- and serotonin-based models [55,56]. Within this variable genus-level profile, the concentration-dependent NO inhibition by the *S. dichotoma* ethyl acetate fraction supports its anti-inflammatory potential without a concurrent loss of RAW 264.7 viability.

The chemical composition of the ethyl acetate fraction is consistent with its NO-inhibitory activity. Quercetin has been reported to inhibit inflammatory cytokines including TNF-α, IL-8, and IL-1α, suppress ROS formation, reduce activation of nuclear factor kappa B (NF- κB) and activator protein 1 (AP-1), and prevent IκBα degradation [46]. Isorhamnetin, the 3′-O-methyl derivative of quercetin, has shown anti-inflammatory effects through inhibition of NO production, inducible NO synthase expression, and mitogen-activated protein kinase (MAPK)-NF-κB signaling [40]. Isorhamnetin 3-O-galactoside and related glycosides have been associated with inhibition of high mobility group box 1 (HMGB1) release and HMGB1-dependent inflammatory responses, as well as reduced HMGB1 receptor expression, NF-κB activation, and TNF-α production [41]. The phytoecdysteroid-rich character of the genus may also contribute to the activity [11]. The co-occurrence of these constituents in the most active fraction therefore agrees with the cellular NO data.

In HaCaT cells, the extract and fractions were non-cytotoxic when applied alone, with viability remaining above 89.29%. In contrast, H_2_O_2_ reduced viability to 45.02%, and co-treatment with the samples did not produce significant overall cytoprotection (*p* > 0.05). The *n*-hexane fraction produced a limited numerical increase to 67.77% at 200 µg/mL, whereas the 80% ethanol extract and water fraction further reduced viability at high concentrations. Minoxidil also failed to prevent H_2_O_2_-induced toxicity. This lack of extract-level cytoprotection contrasts with reports for the isolated flavonols. Isorhamnetin has been shown in HaCaT cells to increase viability, decrease malondialdehyde, enhance superoxide dismutase activity, and inhibit apoptosis during H_2_O_2_-induced oxidative injury [57]. Quercetin has likewise been reported to suppress early apoptotic death and mitochondrial damage in human keratinocytes exposed to H_2_O_2_ [58]. A recent study on *S. baccifera* further demonstrated that its ethanol extract reduced H_2_O_2_-induced ROS accumulation in HaCaT cells in a concentration-dependent manner, accompanied by activation of nuclear factor erythroid 2-related factor 2 (NRF2)-associated antioxidant defenses [59].

Neither minoxidil nor the tested fractions significantly improved viability in the simultaneous H_2_O_2_ co-treatment model. As no treatment produced a positive cytoprotective response in this assay, its ability to detect protection under these conditions was not independently demonstrated. Conclusions on the absence of cytoprotection are limited to the concentrations and exposure schedule studied here.

Taken together, these findings indicate a sample- and concentration-dependent cellular response to oxidative challenge rather than a consistent cytoprotective effect. Because viability measurements alone may not capture the broader metabolic consequences of H_2_O_2_ exposure, metabolomic profiling was subsequently performed to characterize the associated cellular metabolic alterations. At the reported 400 µg/mL condition, the *n*-hexane fraction yielded 38.67% viability during H_2_O_2_ co-treatment, whereas the reported 200 µg/mL condition used for metabolomics yielded a numerical 67.77% viability without statistically demonstrated protection. The higher-dose result cannot be attributed to intrinsic fraction cytotoxicity from the co-treatment comparison alone; it does caution against assuming protection across doses.

The exploratory pairwise results suggest treatment-associated variation in energy-related, redox-associated, nucleotide, amino-acid, and lipid-associated features. Lower ATP and fructose 6-phosphate intensities in the H_2_O_2_ comparison highlight energy-related metabolites as candidates for targeted follow-up. The co-treatment profiles reflect simultaneous fraction and H_2_O_2_ exposure and therefore address responses during oxidative challenge rather than recovery after established injury.

The *n*-hexane and EtOAc groups showed nominal shifts in several central carbon and lipid-associated features. In the *n*-hexane group, the feature putatively annotated as S-hydroxymethylglutathione showed lower intensity. S-hydroxymethylglutathione is a glutathione–formaldehyde adduct involved in ADH5-dependent formaldehyde clearance [60]. This biochemical context may guide targeted follow-up, but formaldehyde production and ADH5 activity were not measured, and a contribution from sample handling remains unresolved.

The co-treatment patterns did not show a consistent shift toward control intensities. The feature putatively annotated as 6-ketoprostaglandin E1 had higher intensity in the EtOAc + H_2_O_2_ group than in the H_2_O_2_-only group.

Minoxidil showed the largest number of exploratory candidates relative to control among the three treatment-only groups. As minoxidil was profiled without H_2_O_2_ and is not a mechanistically matched antioxidant control, this comparison describes differences in metabolomic profiles rather than relative efficacy under oxidative stress.

The overall seven-group analysis detected BH-adjusted differences for 38 of 75 eligible metabolites, indicating variation across the experimental conditions without resolving individual contrasts. No statistically significant pathway enrichment is established by the descriptive KEGG network. Together, these profiles identify candidates for confirmatory measurement and functional follow-up.

Interpretation is limited by three biological replicates per group, missing observations for some metabolites, and unadjusted pairwise testing. Metabolite identities remain putative because MS/MS confirmation was not performed and no annotation reached MSI Level 1; Level 3 assignments, particularly lipid mediators, require confirmation before metabolite-specific mechanisms can be inferred. The combined-treatment groups of the viability and metabolomic experiments involved simultaneous exposure to the fractions and H_2_O_2_. This design did not assess pretreatment-dependent protection or recovery following established oxidative injury and cannot distinguish intracellular protective responses from possible direct chemical interactions between the fractions and H_2_O_2_ in the culture medium. The findings therefore describe responses during co-exposure and do not establish classical cytoprotective mechanisms. The use of a single keratinocyte cell line further limits generalization. In addition, metabolite intensity changes may reflect uptake or residual fraction constituents as well as endogenous metabolism. Additional biological replicates, targeted identity confirmation and functional measurements are needed to test the hypotheses generated here.

## 5. Conclusions

This study provides an integrated phytochemical and cell-based characterization of *Silene dichotoma* Ehrh. subsp. *dichotoma*. The ethyl acetate fraction showed the highest phenolic and flavonoid contents, as well as the strongest antioxidant activity and most pronounced concentration-dependent inhibition of LPS-induced NO production among the tested samples. Bioactivity-guided phytochemical investigation of this fraction yielded isorhamnetin, isorhamnetin-3-O-β-D-galactopyranoside, and quercetin, which, to our knowledge, are reported here for the first time from *S. dichotoma* Ehrh. subsp. *dichotoma*. Although the extract and fractions were non-cytotoxic under the tested conditions, neither they nor the minoxidil comparator significantly protected HaCaT cells against H_2_O_2_-induced cytotoxicity. Metabolomic profiling suggested exploratory treatment-associated patterns in energy-related and other cellular metabolites, including putative lipid-associated features, despite the absence of significant cytoprotection under the tested conditions. Overall, these findings support *S. dichotoma* as a promising source of phenolic and flavonoid constituents with antioxidant and NO-inhibitory potential, while emphasizing the context-dependent nature of its cellular effects. Further investigation using isolated compounds, complementary cytoprotection models, additional cellular systems, and in vivo approaches is warranted to clarify their biological relevance and potential applications.

## Figures and Tables

**Table 1:** Total phenolic and flavonoid contents of the extract and sub-fractions of *S. dichotoma* Ehrh. subsp. *dichotoma*. Values are expressed as mean ± SEM.

Sample	Total Phenolic Content (mg GAE/g Dry Extract)	Total Flavonoid Content (mg QE/g Dry Extract)
80% Ethanol extract	25.52 ± 0.03	37.27 ± 1.91
*n*-Hexane fraction	40.59 ± 0.01	70.77 ± 1.75
Ethyl acetate fraction	51.84 ± 0.24	176.56 ± 10.84
*n*-Butanol fraction	33.34 ± 0.34	29.91 ± 3.22
Water fraction	12.27 ± 0.42	9.92 ± 1.51

**Table 2:** IC_50_ values of the extract and sub-fractions for DPPH radical scavenging activity. Values are expressed as mean ± SEM.

Sample	IC_50_ (µg/mL)
80% Ethanol extract	413.74 ± 36.37
*n*-Hexane fraction	471.82 ± 33.94
Ethyl acetate fraction	180.48 ± 11.19
*n*-Butanol fraction	348.44 ± 25.78
Water fraction	655.57 ± 27.46

## Data Availability

Dataset available on request from the authors.

## Supplementary Materials

The following supporting information can be downloaded at https://www.mdpi.com/article/10.3390/metabo16100765/s1. Table S1: Metabolite-specific MSI identification levels; Table S2: Overall Welch ANOVA of 75 metabolites measured in at least two independent biological replicates in each of seven groups; Table S3: Metabolites meeting exploratory criteria between the H_2_O_2_ and Control groups (unadjusted *p* < 0.05; FC ≥ 1.5 or ≤0.67); Table S4: Metabolites meeting exploratory criteria between the *n*-Hexane Fr. and Control groups (unadjusted *p* < 0.05; FC ≥ 1.5 or ≤0.67); Table S5: Exploratory metabolites with VIP > 1 from the PLS-DA analysis of the *n*-Hexane fraction four-group comparison (Control, H_2_O_2_, *n*-Hexane Fr., *n*-Hexane Fr. + H_2_O_2_); Table S6: Metabolites meeting exploratory criteria between the *n*-Hexane Fr. + H_2_O_2_ and H_2_O_2_ groups (unadjusted *p* < 0.05; FC ≥ 1.5 or ≤0.67); Table S7: Metabolites meeting exploratory criteria between the EtOAc Fr. and Control groups (unadjusted *p* < 0.05; FC ≥ 1.5 or ≤0.67); Table S8: Exploratory metabolites with VIP > 1 from the PLS-DA analysis of the ethyl acetate fraction four-group comparison (Control, H_2_O_2_, EtOAc Fr., EtOAc Fr. + H_2_O_2_); Table S9: Metabolites meeting exploratory criteria between the EtOAc Fr. + H_2_O_2_ and H_2_O_2_ groups (unadjusted *p* < 0.05; FC ≥ 1.5 or ≤0.67); Table S10: Metabolites meeting exploratory criteria between the Minoxidil and Control groups (unadjusted *p* < 0.05; FC ≥ 1.5 or ≤0.67); Table S11: Exploratory metabolites with VIP > 1 from the PLS-DA analysis of the minoxidil comparative four-group analysis (Control, Minoxidil, EtOAc Fr., *n*-Hexane Fr.); Table S12: Metabolites meeting exploratory criteria between the Minoxidil and *n*-Hexane Fr. groups (unadjusted *p* < 0.05; FC ≥ 1.5 or ≤0.67); Table S13: Metabolites meeting exploratory criteria between the Minoxidil and EtOAc Fr. groups (unadjusted *p* < 0.05; FC ≥ 1.5 or ≤0.67); Figure S1: Descriptive heatmap of Control, H_2_O_2_, *n*-Hexane Fr., *n*-Hexane Fr. + H_2_O_2_. The 64 complete-case metabolites were clustered using average linkage and Euclidean distance after log_2_ transformation and row-wise z-scoring across the displayed samples. Color saturation corresponds to z-scores ≤ −2 or ≥+2. Each column represents one biological replicate after averaging analytical injections (n = 3 per group); colors and clusters do not show statistical significance.; Figure S2: Descriptive heatmap of Control, H_2_O_2_, EtOAc Fr., EtOAc Fr. + H_2_O_2_. The 64 complete-case metabolites were clustered using average linkage and Euclidean distance after log_2_ transformation and row-wise z-scoring across the displayed samples. Color saturation corresponds to z-scores ≤ −2 or ≥+2. Each column represents one biological replicate after averaging analytical injections (n = 3 per group); colors and clusters do not show statistical significance.; Figure S3: Descriptive heatmap of Control, Minoxidil, EtOAc Fr., *n*-Hexane Fr. The 64 complete-case metabolites were clustered using average linkage and Euclidean distance after log_2_ transformation and row-wise z-scoring across the displayed samples. Color saturation corresponds to z-scores ≤ −2 or ≥+2. Each column represents one biological replicate after averaging analytical injections (n = 3 per group); colors and clusters do not show statistical significance; Figure S4: Descriptive heatmap of Control, H_2_O_2_, *n*-Hexane Fr., *n*-Hexane Fr. + H_2_O_2_, EtOAc Fr., EtOAc Fr. + H_2_O_2_, Minoxidil. The 64 complete-case metabolites were clustered using average linkage and Euclidean distance after log_2_ transformation and row-wise z-scoring across the displayed samples. Color saturation corresponds to z-scores ≤ −2 or ≥+2. Each column represents one biological replicate after averaging analytical injections (n = 3 per group); colors and clusters do not show statistical significance.

## Abbreviations

The following abbreviations are used in this manuscript:

ABTS	2,2′-Azino-bis(3-ethylbenzothiazoline-6-sulfonic acid)
ANOVA	Analysis of variance
AP-1	Activator protein 1
ATP	Adenosine triphosphate
BH	Benjamini–Hochberg
CCL-18	C-C motif chemokine ligand 18
COX-2	Cyclooxygenase-2
CUPRAC	Cupric ion reducing antioxidant capacity
DMEM	Dulbecco’s Modified Eagle Medium
DMSO	Dimethyl sulfoxide
DPPH	2,2-Diphenyl-1-picrylhydrazyl
EDTAE	Ethylenediaminetetraacetic acid equivalent
EtOAc	Ethyl acetate
FBS	Fetal bovine serum
FC	Fold change
FRAP	Ferric reducing antioxidant power
GAE	Gallic acid equivalent
HaCaT	Human immortalized keratinocyte cell line
HMDB	Human Metabolome Database
HMGB1	High mobility group box 1
HPLC	High-performance liquid chromatography
IC_50_	Half-maximal inhibitory concentration
IL	Interleukin
KEGG	Kyoto Encyclopedia of Genes and Genomes
LC/MS	Liquid chromatography/mass spectrometry
LPS	Lipopolysaccharide
MAPK	Mitogen-activated protein kinase
MSI	Metabolomics Standards Initiative
MTT	3-(4,5-Dimethylthiazol-2-yl)-2,5-diphenyltetrazolium bromide
NAD	Nicotinamide adenine dinucleotide
NBT	Nitroblue tetrazolium
NF-κB	Nuclear factor kappa-light-chain-enhancer of activated B cells
NMR	Nuclear magnetic resonance
NO	Nitric oxide
NRF2	Nuclear factor erythroid 2-related factor 2
PBS	Phosphate-buffered saline
PC	Principal component
PCA	Principal component analysis
PDA	Photodiode array
PLS-DA	Partial least squares discriminant analysis
QE	Quercetin equivalent
Q-TOF	Quadrupole time-of-flight
ROS	Reactive oxygen species
SDS	Sodium dodecyl sulfate
SO	Superoxide
TCA	Tricarboxylic acid (cycle)
TE	Trolox equivalent
TNF-α	Tumor necrosis factor alpha
VIP	Variable importance in projection
VLC	Vacuum liquid chromatography
