# Supplementary material for: Antioxidant and Anti-Inflammatory Activities of Silene dichotoma Ehrh. subsp. dichotoma: Phytochemical Characterization and Metabolomic Insights into Oxidative Stress-Related Responses in HaCaT Cells
Source: Metabolites. 2026 Oct 12;16(10):765. doi: 10.3390/metabo16100765 (PMC13707269; doi:10.3390/metabo16100765)
Supplement: Supplementary file 1 [file metabolites-16-00765-s001.zip › metabolites-4596182-supplementary.pdf]

Supplementary Materials

Antioxidant and Anti-Inflammatory Activities of Silene dichotoma Ehrh. subsp. dichotoma: Phytochemical Characterization and Metabolomic Insights into Oxidative Stress-Related Responses in HaCaT Cells

Melek Nur Bilal, Ozan Kaplan, Mustafa Çelebier and Vahap Murat Kutluay *

* Correspondence: muratkutluay@hacettepe.edu.tr

Manuscript ID: metabolites-4596182

Contents

Table S1. MSI annotation levels (99 annotations).

Table S2. Overall seven-group Welch ANOVA (75 eligible metabolites).

Tables S3–S4. H_2O_2 and n-hexane fraction versus Control.

Table S5. VIP rankings for the n-hexane four-group model.

Table S6. n-Hexane fraction + H_2O_2 versus H_2O_2.

Table S7. EtOAc fraction versus Control.

Table S8. VIP rankings for the EtOAc four-group model.

Table S9. EtOAc fraction + H_2O_2 versus H_2O_2.

Table S10. Minoxidil versus Control.

Table S11. VIP rankings for the minoxidil comparative model.

Tables S12–S13. Minoxidil versus n-hexane and EtOAc fractions.

Figures S1–S4. Heatmaps at the biological-replicate level.

Analysis and interpretation

Paired analytical injections were averaged within biological samples, yielding three biological replicates per group. When only one injection yielded a signal, that value represented the sample; when both were missing, the sample value remained missing. Missing values were not imputed. Univariate tests required at least two valid biological replicates in every group included in the comparison.

Table S2 reports Benjamini–Hochberg-adjusted q values for the overall comparison. Pairwise tables report exploratory candidates selected using unadjusted $p < 0.05$ and FC ≥ 1.5 or ≤ 0.67. VIP rankings and heatmap patterns are descriptive. Annotation confidence is defined in Table S1. EtOAc, ethyl acetate; Fr., fraction; FC, fold change; VIP, variable importance in projection.

Table S1. Metabolite-specific MSI identification levels.

Metabolite	MSI Level
Adenosine triphosphate	Level 2
NAD	Level 2
Glutamic acid	Level 2
D-Glucose	Level 3
Succinic acid	Level 2
Lysine	Level 2
L-Aspartic acid	Level 2
Glutathione	Level 3
Adenosine 3',5'-diphosphate	Level 2
Cytidine monophosphate	Level 2
L-Arginine	Level 2
Glutamine	Level 2
Serine	Level 2
Ornithine	Level 2
L-Tryptophan	Level 3
Phenylalanine	Level 2
L-Tyrosine	Level 2
Fructose 6-phosphate	Level 2
Glucose 6-phosphate	Level 2
2-Ketobutyric acid	Level 3
Dihydroxyacetone phosphate	Level 2
D-Ribose 5-phosphate	Level 2
Fumaric acid	Level 2
Leucine	Level 2
Histidine	Level 2
Adenine	Level 2
Proline	Level 2
Malic acid	Level 3
Citric acid	Level 2
L-Valine	Level 2
Dihydroxyacetone	Level 3
L-Threonine	Level 2
Oxalic acid	Level 2
Adenosine	Level 2
Thymidine	Level 2
Ketoleucine	Level 3
dCMP	Level 3
Guanine	Level 3
Taurine	Level 2
Palmitic acid	Level 3
Orotic acid	Level 2
Uridine	Level 2
Deoxyguanosine	Level 2
gamma-Aminobutyric acid	Level 2
4,5-Dihydroorotic acid	Level 2
5-Thymidylic acid	Level 2
dUMP	Level 2
Uric acid	Level 2
N2-Acetylornithine	Level 3
Nicotinamide ribotide	Level 3
Cytidine	Level 2
D-Malic acid	Level 3
Homogentisic acid	Level 3
Cyclic AMP	Level 2
N-Acetyl-L-glutamic acid	Level 2
3-Hydroxyanthranilic acid	Level 2
Oleic acid	Level 3
all-trans-Retinoic acid	Level 3
Mesoxalic acid	Level 2
Leukotriene A4	Level 3

Metabolite	MSI Level
3-Mercaptopyruvic acid	Level 3
Prostaglandin B1	Level 3
S-Acetyldihydrolipoamide	Level 3
3,4-Dihydroxybenzeneacetic acid	Level 2
7-Dehydrocholesterol	Level 3
Glycerylphosphorylethanolamine	Level 3
Diadenosine tetraphosphate	Level 3
myo-Inositol 1,3,4,5-tetrakisphosphate	Level 3
2'-Deoxyinosine triphosphate	Level 3
Stearic acid	Level 3
Linoleic acid	Level 3
Galactitol	Level 2
Pyroglutamic acid	Level 2
3-maleylpyruvic acid	Level 3
Methylmalonic acid	Level 2
Xanthurenic acid	Level 2
Dodecanoic acid	Level 3
beta-Glycerophosphoric acid	Level 3
cis-4-Hydroxy-D-proline	Level 2
3,4-Dihydroxyphenylacetaldehyde	Level 3
D-myo-Inositol 1,3,4,6-tetrakisphosphate	Level 3
D-myo-Inositol 3,4,5,6-tetrakisphosphate	Level 3
L-Xylonic acid	Level 3
L-Lyxonic acid	Level 3
Biocytin	Level 3
Formylanthranilic acid	Level 3
gamma-Glutamyl-beta-cyanoalanine (putative)	Level 3
6-Ketoprostaglandin E1	Level 3
6-Hydroxyhexanoic acid	Level 3
Caprylic acid	Level 3
Myristic acid	Level 3
Azelaic acid	Level 3
D-myo-Inositol 1,4,5,6-tetrakisphosphate	Level 3
MG(0:0/20:4(5Z,8Z,11Z,14Z)/0:0)	Level 3
S-Hydroxymethylglutathione	Level 3
Purine	Level 2
LysoPA(P-16:0/0:0)	Level 3
S-Acetyldihydrolipoamide-E	Level 3
N-Acetylneuraminic acid	Level 2

Identification levels follow the Metabolomics Standards Initiative (MSI) recommendations. Level 2: putatively annotated compound (match to an authentic reference standard of the in-house library, analyzed under identical LC–QTOF–MS conditions, on the basis of accurate mass and retention time; MS/MS spectra were not acquired); Level 3: putatively characterized compound (accurate-mass match against public databases, HMDB and METLIN). No metabolite reached MSI Level 1; all metabolite names should therefore be regarded as putative annotations. γ-Glutamyl-β-cyanoalanine is not an established mammalian metabolite and was detected in all experimental groups, including the untreated control; it is reported throughout the Supplementary Tables as a putative annotation only and was not interpreted in the main text.

Table S2. Overall Welch ANOVA of 75 metabolites measured in at least two independent biological replicates in each of seven groups.

Metabolite	Welch F	Raw p	BH q
S-Hydroxymethylglutathione	235.342	1.91e-06	0.000143
gamma-Aminobutyric acid	73.535	4.23e-05	0.000987
MG(0:0/20:4(5Z,8Z,11Z,14Z)/0:0)	62.185	5.26e-05	0.000987
3-maleylpyruvic acid	73.233	5.96e-05	0.000987
L-Threonine	57.246	6.58e-05	0.000987
beta-Glycerophosphoric acid	74.238	0.000101	0.0010
Adenosine triphosphate	64.870	0.00011	0.0010
cis-4-Hydroxy-D-proline	73.438	0.000111	0.0010
Citric acid	40.855	0.000139	0.0011
N-Acetylneuraminic acid	38.396	0.000144	0.0011
L-Tyrosine	36.536	0.000163	0.0011
Adenine	36.474	0.000197	0.0011
dCMP	40.099	0.000213	0.0011
Deoxyguanosine	34.691	0.000215	0.0011
gamma-Glutamyl-beta-cyanoalanine	40.765	0.000228	0.0011
Glutamic acid	34.227	0.000334	0.0016
Fructose 6-phosphate	29.008	0.000483	0.0021
Galactitol	23.203	0.000589	0.0023
Azelaic acid	24.033	0.000592	0.0023
Xanthurenic acid	23.577	0.000601	0.0023
Taurine	25.706	0.000738	0.0026
L-Aspartic acid	22.787	0.000787	0.0027
Adenosine	24.316	0.000912	0.0030
D-Malic acid	18.789	0.0013	0.0041
L-Xylonic acid	19.601	0.0014	0.0041
Fumaric acid	15.663	0.0019	0.0055
Malic acid	15.252	0.0021	0.0059
Adenosine 3',5'-diphosphate	13.727	0.0034	0.0089
Oleic acid	12.392	0.0035	0.0089
L-Tryptophan	11.335	0.0048	0.0117
dUMP	10.799	0.0049	0.0117
S-Acetyldihydrolipoamide	11.594	0.0052	0.0122
Glutamine	7.636	0.0139	0.0316
L-Lyxonic acid	18.638	0.0146	0.0322
myo-Inositol 1,3,4,5-tetrakisphosphate	6.773	0.0173	0.0370
Stearic acid	5.774	0.0243	0.0495
Dodecanoic acid	8.355	0.0249	0.0495
2-Ketobutyric acid	6.033	0.0251	0.0495
Leucine	5.259	0.0351	0.0675
Uridine	4.882	0.0428	0.0803
Linoleic acid	4.168	0.0555	0.1016
Dihydroxyacetone phosphate	3.871	0.0598	0.1068
6-Ketoprostaglandin E1	3.772	0.0653	0.1116
Histidine	4.773	0.0655	0.1116
Serine	3.562	0.0787	0.1311
Orotic acid	3.458	0.0853	0.1381
Mesoxalic acid	3.382	0.0865	0.1381
D-myo-Inositol 3,4,5,6-tetrakisphosphate	3.193	0.0898	0.1404
Palmitic acid	2.934	0.1094	0.1674
Ornithine	2.866	0.1123	0.1684
Cyclic AMP	2.495	0.1520	0.2235
3,4-Dihydroxyphenylacetaldehyde	2.701	0.1695	0.2426
Uric acid	2.244	0.1728	0.2426
4,5-Dihydroorotic acid	2.219	0.1747	0.2426
Myristic acid	1.999	0.2267	0.3092
D-myo-Inositol 1,3,4,6-tetrakisphosphate	1.786	0.2495	0.3342
3-Mercaptopyruvic acid	1.713	0.2769	0.3644
Thymidine	1.438	0.3370	0.4305
Guanine	1.513	0.3387	0.4305

Metabolite	Welch F	Raw p	BH q
Methylmalonic acid	1.329	0.3670	0.4588
N2-Acetylornithine	1.283	0.3844	0.4726
6-Hydroxyhexanoic acid	1.233	0.4026	0.4871
Phenylalanine	1.192	0.4238	0.5046
L-Arginine	0.872	0.5650	0.6621
Succinic acid	0.637	0.7012	0.8091
Formylanthranilic acid	0.594	0.7285	0.8193
D-Glucose	0.589	0.7319	0.8193
Dihydroxyacetone	0.528	0.7721	0.8516
Oxalic acid	0.405	0.8518	0.9254
Lysine	0.387	0.8637	0.9254
Pyroglutamic acid	0.329	0.8975	0.9481
3,4-Dihydroxybenzeneacetic acid	0.309	0.9112	0.9491
Leukotriene A4	0.267	0.9272	0.9527
Homogentisic acid	0.149	0.9818	0.9854
D-myo-Inositol 1,4,5,6-tetrakisphosphate	0.137	0.9854	0.9854

Paired analytical injections were averaged within each independent biological replicate (at most three per group). The 75 metabolites detected in at least two biological replicates in every group were analyzed with Welch one-way ANOVA after $\log_2$ transformation. Missing values were not imputed. Benjamini–Hochberg correction was applied across these 75 tests; an overall $q < 0.05$ does not identify the differing group pairs.

Table S3. Metabolites meeting exploratory criteria between the H_2O_2 and Control groups (unadjusted $p < 0.05$; FC ≥ 1.5 or ≤ 0.67).

Metabolite	Reference mean	Treatment mean	n ref/trt	FC	Unadjusted p	Direction
Fructose 6-phosphate	75,862.7	36,393.8	3/3	0.480	0.0074	↓
Adenosine triphosphate	15,814.0	10,110.4	3/3	0.639	0.0076	↓
beta-Glycerophosphoric acid	12,238.0	5,675.0	3/3	0.464	0.0095	↓
5-Thymidylic acid	6,221.6	1,513.9	3/2	0.243	0.0141	↓
Adenine	10,165.5	18,662.3	3/3	1.836	0.0188	↑
dUMP	28,156.5	14,622.1	3/3	0.519	0.0249	↓
S-Hydroxymethylglutathione	193,964.6	101,612.9	3/3	0.524	0.0260	↓
Uric acid	20,204.6	36,157.3	3/3	1.790	0.0425	↑
Guanine	4,846.2	1,643.5	3/3	0.339	0.0493	↓

Reference: Control; treatment: H_2O_2. Means are untransformed cell-count-normalized signal intensities; n ref/trt gives the number of valid biological replicates. FC = treatment mean/reference mean. Two-sided Welch t-tests used $\log_2$-transformed intensities. ↑ and ↓ indicate higher and lower mean intensity, respectively. The p values are unadjusted; these candidates are exploratory.

Table S4. Metabolites meeting exploratory criteria between the n-Hexane Fr. and Control groups (unadjusted $p < 0.05$; FC ≥ 1.5 or ≤ 0.67).

Metabolite	Reference mean	Treatment mean	n ref/trt	FC	Unadjusted p	Direction
MG(0:0/20:4(5Z,8Z,11Z,14Z)/0:0)	6,851.9	3,873.8	3/3	0.565	5.71e-05	↓
Citric acid	17,037.6	1,182.5	3/3	0.069	0.000768	↓
S-Hydroxymethylglutathione	193,964.6	19,773.5	3/3	0.102	0.000777	↓
N-Acetylneuraminic acid	46,769.9	3,015.3	3/3	0.064	0.000782	↓
beta-Glycerophosphoric acid	12,238.0	1,094.1	3/2	0.089	0.0010	↓
dUMP	28,156.5	4,696.2	3/3	0.167	0.0013	↓
gamma-Aminobutyric acid	103,160.8	11,740.0	3/3	0.114	0.0019	↓
L-Aspartic acid	21,145.8	2,011.5	3/3	0.095	0.0061	↓
Taurine	324,941.6	36,161.1	3/3	0.111	0.0075	↓
3-maleylpyruvic acid	28,643.2	10,368.1	3/3	0.362	0.0089	↓
Glutamic acid	195,729.1	38,899.8	3/3	0.199	0.0117	↓
Adenine	10,165.5	5,289.1	3/3	0.520	0.0120	↓
Fumaric acid	25,466.2	52,000.0	3/3	2.042	0.0157	↑
Adenosine	6,847.9	2,188.3	3/3	0.320	0.0160	↓
4,5-Dihydroorotic acid	18,191.3	7,458.5	3/3	0.410	0.0222	↓
Fructose 6-phosphate	75,862.7	4,591.3	3/3	0.061	0.0241	↓
Linoleic acid	21,948.7	96,470.9	3/3	4.395	0.0272	↑
Dodecanoic acid	1,523.6	9,810.3	2/3	6.439	0.0347	↑
7-Dehydrocholesterol	4,397.9	15,987.1	3/2	3.635	0.0358	↑
dCMP	8,051.5	47,086.6	3/3	5.848	0.0393	↑
Cyclic AMP	25,936.8	13,971.1	3/3	0.539	0.0395	↓
gamma-Glutamyl-beta-cyanoalanine	3,377.1	6,942.7	3/3	2.056	0.0401	↑
Adenosine triphosphate	15,814.0	1,395.0	3/2	0.088	0.0414	↓
3,4-Dihydroxyphenylacetaldehyde	1,837.8	1,178.4	3/3	0.641	0.0426	↓
Mesoxalic acid	8,905.8	3,765.1	3/3	0.423	0.0444	↓

Reference: Control; treatment: n-Hexane Fr. Means are untransformed cell-count-normalized signal intensities; n ref/trt gives the number of valid biological replicates. FC = treatment mean/reference mean. Two-sided Welch t-tests used $\log_2$-transformed intensities. ↑ and ↓ indicate higher and lower mean intensity, respectively. The p values are unadjusted; these candidates are exploratory.

Table S5. Exploratory metabolites with VIP > 1 from the PLS-DA analysis of the n-Hexane fraction four-group comparison (Control, H_2O_2, n-Hexane Fr., n-Hexane Fr. + H_2O_2).

Metabolite	VIP
Adenine	1.740
Azelaic acid	1.624
Mesoxalic acid	1.502
Oleic acid	1.422
Fumaric acid	1.411
Methylmalonic acid	1.392
Stearic acid	1.386
L-Tyrosine	1.330
Dihydroxyacetone phosphate	1.274
L-Aspartic acid	1.272
Leucine	1.261
Citric acid	1.236
6-Ketoprostaglandin E1	1.221
S-Hydroxymethylglutathione	1.217
L-Xylonic acid	1.214
Cyclic AMP	1.192
3-maleylpyruvic acid	1.180
N-Acetylneuraminic acid	1.175
Xanthurenic acid	1.164
Glutamic acid	1.164
L-Tryptophan	1.162
Fructose 6-phosphate	1.152
dUMP	1.152
Adenosine 3',5'-diphosphate	1.137
gamma-Aminobutyric acid	1.133
Palmitic acid	1.128
L-Threonine	1.110
D-Malic acid	1.088
Taurine	1.086
Deoxyguanosine	1.067
MG(0:0/20:4(5Z,8Z,11Z,14Z)/0:0)	1.046
Malic acid	1.030
Phenylalanine	1.023
Linoleic acid	1.007

VIP > 1 values are shown for the two-component PLS-DA model fitted to 64 complete-case metabolites, with three biological replicates per group. VIP measures variable contribution to the fitted model and is not a metabolite-level significance test. Model validation is reported in Section 3.7.6 of the main manuscript.

Table S6. Metabolites meeting exploratory criteria between the n-Hexane Fr. + H_2O_2 and H_2O_2 groups (unadjusted p < 0.05; FC ≥ 1.5 or ≤ 0.67).

Metabolite	Reference mean	Treatment mean	n ref/trt	FC	Unadjusted p	Direction
Azelaic acid	84,782.1	42,999.7	3/3	0.507	0.000151	↓
Adenine	18,662.3	4,894.9	3/3	0.262	0.000244	↓
Deoxyguanosine	6,766.1	3,052.7	3/3	0.451	0.0041	↓
Oleic acid	8,302.2	36,196.7	3/3	4.360	0.0067	↑
Stearic acid	5,820.6	3,460.7	3/3	0.595	0.0089	↓
7-Dehydrocholesterol	2,309.7	7,003.7	2/3	3.032	0.0158	↑
L-Aspartic acid	16,965.8	5,883.3	3/3	0.347	0.0174	↓
Citric acid	12,670.6	4,894.7	3/3	0.386	0.0415	↓

Reference: H_2O_2; treatment: n-Hexane Fr. + H_2O_2. Means are untransformed cell-count-normalized signal intensities; n ref/trt gives the number of valid biological replicates. FC = treatment mean/reference mean. Two-sided Welch t-tests used $\log_2$-transformed intensities. ↑ and ↓ indicate higher and lower mean intensity, respectively. The p values are unadjusted; these candidates are exploratory.

Table S7. Metabolites meeting exploratory criteria between the EtOAc Fr. and Control groups (unadjusted p < 0.05; FC ≥ 1.5 or ≤ 0.67).

Metabolite	Reference mean	Treatment mean	n ref/trt	FC	Unadjusted p	Direction
Adenosine triphosphate	15,814.0	1,413.0	3/3	0.089	3.08e-05	↓
N-Acetylneuraminic acid	46,769.9	3,403.2	3/3	0.073	8.06e-05	↓
NAD	11,363.3	1,074.5	3/2	0.095	0.000131	↓
Glycerylphosphorylethanolamine	26,747.1	1,025.8	3/2	0.038	0.000141	↓
beta-Glycerophosphoric acid	12,238.0	1,135.0	3/2	0.093	0.000454	↓
Cytidine	14,075.4	1,051.6	3/2	0.075	0.000512	↓
Citric acid	17,037.6	1,583.2	3/3	0.093	0.0012	↓
L-Tyrosine	42,804.7	16,942.2	3/3	0.396	0.0025	↓
S-Hydroxymethylglutathione	193,964.6	24,975.2	3/3	0.129	0.0028	↓
3-maleylpyruvic acid	28,643.2	10,460.9	3/3	0.365	0.0033	↓
L-Xylonic acid	57,715.2	5,746.5	3/3	0.100	0.0048	↓
dUMP	28,156.5	5,362.0	3/3	0.190	0.0049	↓
gamma-Aminobutyric acid	103,160.8	17,221.5	3/3	0.167	0.0065	↓
cis-4-Hydroxy-D-proline	8,087.0	1,278.1	3/2	0.158	0.0096	↓
Taurine	324,941.6	47,760.1	3/3	0.147	0.0100	↓
L-Aspartic acid	21,145.8	2,248.9	3/3	0.106	0.0117	↓
Glutamic acid	195,729.1	45,719.6	3/3	0.234	0.0119	↓
L-Threonine	39,263.1	13,658.3	3/3	0.348	0.0162	↓
Fructose 6-phosphate	75,862.7	2,712.2	3/3	0.036	0.0173	↓
D-Malic acid	23,361.9	3,315.7	3/3	0.142	0.0202	↓
dCMP	8,051.5	52,590.7	3/3	6.532	0.0294	↑
Glutathione	9,442.6	1,105.6	3/2	0.117	0.0340	↓
Malic acid	7,002.7	2,223.6	3/3	0.318	0.0341	↓
Xanthurenic acid	31,384.0	13,317.0	3/3	0.424	0.0364	↓
Dodecanoic acid	1,523.6	12,187.9	2/2	8.000	0.0379	↑
gamma-Glutamyl-beta-cyanoalanine	3,377.1	6,536.8	3/3	1.936	0.0382	↑
Serine	16,686.3	6,936.0	3/3	0.416	0.0386	↓
Linoleic acid	21,948.7	79,395.3	3/3	3.617	0.0427	↑

Reference: Control; treatment: EtOAc Fr. Means are untransformed cell-count-normalized signal intensities; n ref/trt gives the number of valid biological replicates. FC = treatment mean/reference mean. Two-sided Welch t-tests used $\log_2$-transformed intensities. ↑ and ↓ indicate higher and lower mean intensity, respectively. The p values are unadjusted; these candidates are exploratory.

Table S8. Exploratory metabolites with VIP > 1 from the PLS-DA analysis of the ethyl acetate fraction four-group comparison (Control, H_2O_2, EtOAc Fr., EtOAc Fr. + H_2O_2).

**Metabolite**	**VIP**
Oleic acid	1.671
Fumaric acid	1.576
L-Tyrosine	1.494
S-Acetyldihydrolipoamide	1.381
L-Tryptophan	1.343
2-Ketobutyric acid	1.275
3-maleylpyruvic acid	1.275
Xanthurenic acid	1.273
dCMP	1.268
L-Threonine	1.261
3-Mercaptopyruvic acid	1.213
Malic acid	1.211
L-Aspartic acid	1.204
Fructose 6-phosphate	1.186
Serine	1.180
Citric acid	1.178
N-Acetylneuraminic acid	1.176
S-Hydroxymethylglutathione	1.175
Glutamic acid	1.165
Dihydroxyacetone	1.152
Phenylalanine	1.148
D-Malic acid	1.144
Taurine	1.137
D-myo-Inositol 3,4,5,6-tetrakisphosphate	1.120
dUMP	1.111
Ornithine	1.100
gamma-Aminobutyric acid	1.086
D-myo-Inositol 1,3,4,6-tetrakisphosphate	1.084
Succinic acid	1.063
Mesoxalic acid	1.041
Deoxyguanosine	1.038
Leucine	1.028

VIP > 1 values are shown for the two-component PLS-DA model fitted to 64 complete-case metabolites, with three biological replicates per group. VIP measures variable contribution to the fitted model and is not a metabolite-level significance test. Model validation is reported in Section 3.7.6 of the main manuscript.

Table S9. Metabolites meeting exploratory criteria between the EtOAc Fr. + H_2O_2 and H_2O_2 groups (unadjusted $p < 0.05$; FC ≥ 1.5 or ≤ 0.67).

Metabolite	Reference mean	Treatment mean	n ref/trt	FC	Unadjusted p	Direction
Oleic acid	8,302.2	27,847.1	3/3	3.354	0.0030	↑
Adenine	18,662.3	11,975.0	3/3	0.642	0.0037	↓
Galactitol	154,381.6	26,915.0	3/3	0.174	0.0044	↓
Glucose 6-phosphate	14,103.1	4,352.1	3/3	0.309	0.0061	↓
L-Tryptophan	22,940.4	37,530.1	3/3	1.636	0.0094	↑
Malic acid	9,845.3	3,310.1	3/3	0.336	0.0099	↓
Deoxyguanosine	6,766.1	2,260.5	3/3	0.334	0.0106	↓
Adenosine triphosphate	10,110.4	6,727.7	3/3	0.665	0.0130	↓
Fructose 6-phosphate	36,393.8	14,840.5	3/3	0.408	0.0143	↓
Uric acid	36,157.3	21,192.1	3/3	0.586	0.0206	↓
Dihydroxyacetone phosphate	5,184.4	2,944.5	3/3	0.568	0.0229	↓
beta-Glycerophosphoric acid	5,675.0	3,323.8	3/3	0.586	0.0323	↓
6-Ketoprostaglandin E1	7,735.9	15,823.6	3/3	2.045	0.0356	↑
S-Acetyldihydrolipoamide	10,412.5	4,460.0	3/3	0.428	0.0426	↓
Phenylalanine	28,506.5	15,736.5	3/3	0.552	0.0481	↓
Biocytin	19,993.8	1,284.0	3/2	0.064	0.0498	↓

Reference: H_2O_2; treatment: EtOAc Fr. + H_2O_2. Means are untransformed cell-count-normalized signal intensities; n ref/trt gives the number of valid biological replicates. FC = treatment mean/reference mean. Two-sided Welch t-tests used $\log_2$-transformed intensities. ↑ and ↓ indicate higher and lower mean intensity, respectively. The p values are unadjusted; these candidates are exploratory.

Table S10. Metabolites meeting exploratory criteria between the Minoxidil and Control groups (unadjusted $p < 0.05$; FC ≥ 1.5 or ≤ 0.67).

Metabolite	Reference mean	Treatment mean	n ref/trt	FC	Unadjusted p	Direction
Ketoleucine	6,952.2	1,100.7	3/3	0.158	8.04e-05	↓
N-Acetylneuraminic acid	46,769.9	6,789.4	3/3	0.145	0.000366	↓
Glucose 6-phosphate	16,631.0	2,718.7	3/3	0.163	0.000831	↓
L-Threonine	39,263.1	9,410.6	3/3	0.240	0.000976	↓
Xanthurenic acid	31,384.0	10,000.6	3/3	0.319	0.0010	↓
Citric acid	17,037.6	2,513.8	3/3	0.148	0.0013	↓
Taurine	324,941.6	32,215.1	3/3	0.099	0.0020	↓
Proline	3,967.0	1,141.7	3/3	0.288	0.0022	↓
D-Malic acid	23,361.9	8,700.1	3/3	0.372	0.0057	↓
S-Hydroxymethylglutathione	193,964.6	40,346.7	3/3	0.208	0.0061	↓
L-Tyrosine	42,804.7	18,597.3	3/3	0.434	0.0070	↓
Nicotinamide ribotide	12,254.6	2,445.8	3/3	0.200	0.0079	↓
3-maleylpyruvic acid	28,643.2	13,768.7	3/3	0.481	0.0096	↓
Adenosine	6,847.9	2,136.3	3/2	0.312	0.0097	↓
Stearic acid	5,700.7	2,101.0	3/3	0.369	0.0098	↓
beta-Glycerophosphoric acid	12,238.0	2,026.6	3/3	0.166	0.0105	↓
dCMP	8,051.5	106,962.9	3/3	13.285	0.0139	↑
Oleic acid	12,255.9	4,034.9	3/3	0.329	0.0145	↓
dUMP	28,156.5	4,514.6	3/3	0.160	0.0166	↓
2-Ketobutyric acid	10,337.4	3,910.9	3/3	0.378	0.0168	↓
gamma-Aminobutyric acid	103,160.8	27,074.2	3/3	0.262	0.0250	↓
Glutamic acid	195,729.1	73,073.8	3/3	0.373	0.0258	↓
MG(0:0/20:4(5Z,8Z,11Z,14Z)/0:0)	6,851.9	3,445.0	3/3	0.503	0.0267	↓
4,5-Dihydroorotic acid	18,191.3	9,958.7	3/3	0.547	0.0291	↓
gamma-Glutamyl-beta-cyanoalanine	3,377.1	8,226.6	3/3	2.436	0.0304	↑
N-Acetyl-L-glutamic acid	5,285.5	1,002.8	3/2	0.190	0.0314	↓
Myristic acid	7,067.9	17,130.4	3/3	2.424	0.0335	↑
Deoxyguanosine	3,323.9	1,161.0	3/3	0.349	0.0357	↓
S-Acetyldihydrolipoamide	8,170.5	2,787.9	3/3	0.341	0.0367	↓
Adenosine triphosphate	15,814.0	3,279.6	3/3	0.207	0.0376	↓
Cyclic AMP	25,936.8	12,845.6	3/3	0.495	0.0389	↓
Linoleic acid	21,948.7	188,187.9	3/3	8.574	0.0413	↑
Cytidine	14,075.4	2,752.1	3/3	0.196	0.0438	↓
Histidine	5,224.9	1,347.9	3/2	0.258	0.0446	↓
Leucine	112,587.1	63,014.7	3/3	0.560	0.0488	↓

Reference: Control; treatment: Minoxidil. Means are untransformed cell-count-normalized signal intensities; n ref/trt gives the number of valid biological replicates. FC = treatment mean/reference mean. Two-sided Welch t-tests used $\log_2$-transformed intensities. ↑ and ↓ indicate higher and lower mean intensity, respectively. The p values are unadjusted; these candidates are exploratory.

Table S11. Exploratory metabolites with VIP > 1 from the PLS-DA analysis of the minoxidil comparative four-group analysis (Control, Minoxidil, EtOAc Fr., n-Hexane Fr.).

Metabolite	VIP
myo-Inositol 1,3,4,5-tetrakisphosphate	1.534
Fructose 6-phosphate	1.511
S-Acetyldihydrolipoamide	1.494
Stearic acid	1.464
Orotic acid	1.421
L-Aspartic acid	1.418
MG(0:0/20:4(5Z,8Z,11Z,14Z)/0:0)	1.409
Uridine	1.367
D-Malic acid	1.336
N-Acetylneuraminic acid	1.306
Glutamic acid	1.303
Dihydroxyacetone phosphate	1.296
S-Hydroxymethylglutathione	1.295
3-maleylpyruvic acid	1.289
dCMP	1.282
gamma-Aminobutyric acid	1.268
Citric acid	1.265
Galactitol	1.260
Taurine	1.246
3-Mercaptopyruvic acid	1.189
gamma-Glutamyl-beta-cyanoalanine	1.186
Linoleic acid	1.179
dUMP	1.174
Deoxyguanosine	1.168
Adenosine 3',5'-diphosphate	1.154
Fumaric acid	1.132
L-Threonine	1.098
Adenine	1.084
Xanthurenic acid	1.076

VIP > 1 values are shown for the two-component PLS-DA model fitted to 64 complete-case metabolites, with three biological replicates per group. VIP measures variable contribution to the fitted model and is not a metabolite-level significance test. Model validation is reported in Section 3.7.6 of the main manuscript.

Table S12. Metabolites meeting exploratory criteria between the Minoxidil and n-Hexane Fr. groups (unadjusted $p < 0.05$; FC ≥ 1.5 or ≤ 0.67).

Metabolite	Reference mean	Treatment mean	n ref/trt	FC	Unadjusted p	Direction
Galactitol	30,532.6	95,024.2	3/3	3.112	0.0091	↑
S-Hydroxymethylglutathione	19,773.5	40,346.7	3/3	2.040	0.0154	↑
Glutamic acid	38,899.8	73,073.8	3/3	1.879	0.0157	↑
dCMP	47,086.6	106,962.9	3/3	2.272	0.0185	↑
L-Aspartic acid	2,011.5	7,889.3	3/3	3.922	0.0351	↑
N-Acetylneuraminic acid	3,015.3	6,789.4	3/3	2.252	0.0356	↑
Citric acid	1,182.5	2,513.8	3/3	2.126	0.0424	↑
Fructose 6-phosphate	4,591.3	24,590.8	3/3	5.356	0.0435	↑

Reference: n-Hexane Fr.; treatment: Minoxidil. Means are untransformed cell-count-normalized signal intensities; n ref/trt gives the number of valid biological replicates. FC = treatment mean/reference mean. Two-sided Welch t-tests used $\log_2$-transformed intensities. ↑ and ↓ indicate higher and lower mean intensity, respectively. The p values are unadjusted; these candidates are exploratory.

Table S13. Metabolites meeting exploratory criteria between the Minoxidil and EtOAc Fr. groups (unadjusted $p < 0.05$; FC $\geq$ 1.5 or ≤ 0.67).

Metabolite	Reference mean	Treatment mean	n ref/trt	FC	Unadjusted p	Direction
Galactitol	13,026.6	95,024.2	3/3	7.295	0.0012	↑
S-Acetyldihydrolipoamide	6,388.4	2,787.9	3/3	0.436	0.0037	↓
N-Acetylneuraminic acid	3,403.2	6,789.4	3/3	1.995	0.0089	↑
dCMP	52,590.7	106,962.9	3/3	2.034	0.0121	↑
Fructose 6-phosphate	2,712.2	24,590.8	3/3	9.067	0.0200	↑
Uridine	2,633.6	1,378.2	3/3	0.523	0.0226	↓
Glutamic acid	45,719.6	73,073.8	3/3	1.598	0.0335	↑
Adenosine 3',5'-diphosphate	5,405.0	10,576.9	3/3	1.957	0.0365	↑
3,4-Dihydroxyphenylacetaldehyde	2,510.5	1,145.6	3/2	0.456	0.0393	↓
L-Lyxonic acid	3,070.1	12,065.8	3/2	3.930	0.0426	↑
MG(0:0/20:4(5Z,8Z,11Z,14Z)/0:0)	5,457.4	3,445.0	3/3	0.631	0.0455	↓
Stearic acid	5,171.8	2,101.0	3/3	0.406	0.0465	↓
L-Aspartic acid	2,248.9	7,889.3	3/3	3.508	0.0473	↑
S-Hydroxymethylglutathione	24,975.2	40,346.7	3/3	1.615	0.0491	↑

Reference: EtOAc Fr.; treatment: Minoxidil. Means are untransformed cell-count-normalized signal intensities; n ref/trt gives the number of valid biological replicates. FC = treatment mean/reference mean. Two-sided Welch t-tests used $\log_2$-transformed intensities. ↑ and ↓ indicate higher and lower mean intensity, respectively. The p values are unadjusted; these candidates are exploratory.

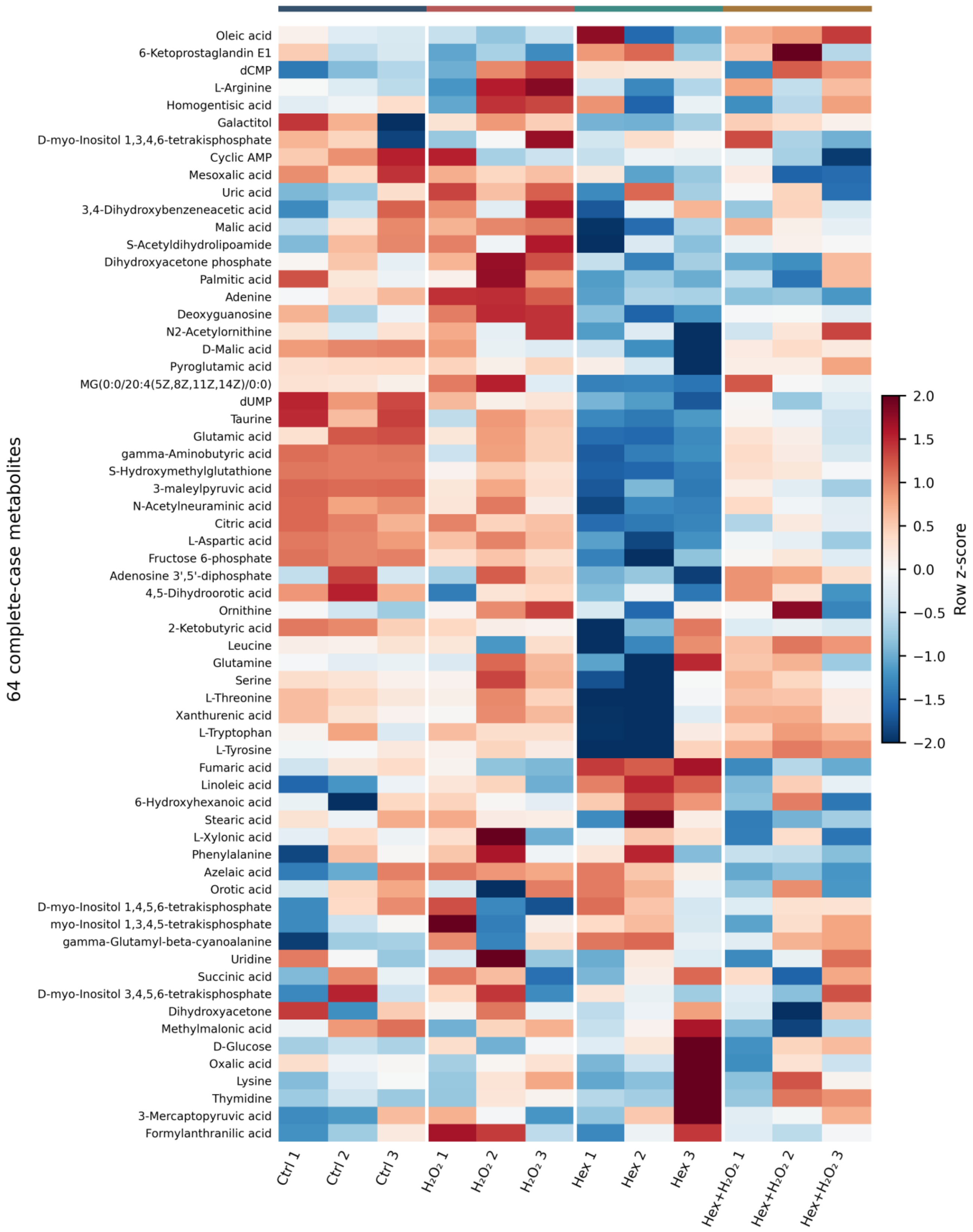

Figure S1. Descriptive heatmap of Control, H_2O_2, n-Hexane Fr., n-Hexane Fr. + H_2O_2. The 64 complete-case metabolites were clustered using average linkage and Euclidean distance after $\log_2$ transformation and row-wise z-scoring across the displayed samples. Color saturation corresponds to z-scores ≤ -2 or $\geq +2$. Each column represents one biological replicate after averaging analytical injections (n = 3 per group); colors and clusters do not show statistical significance.

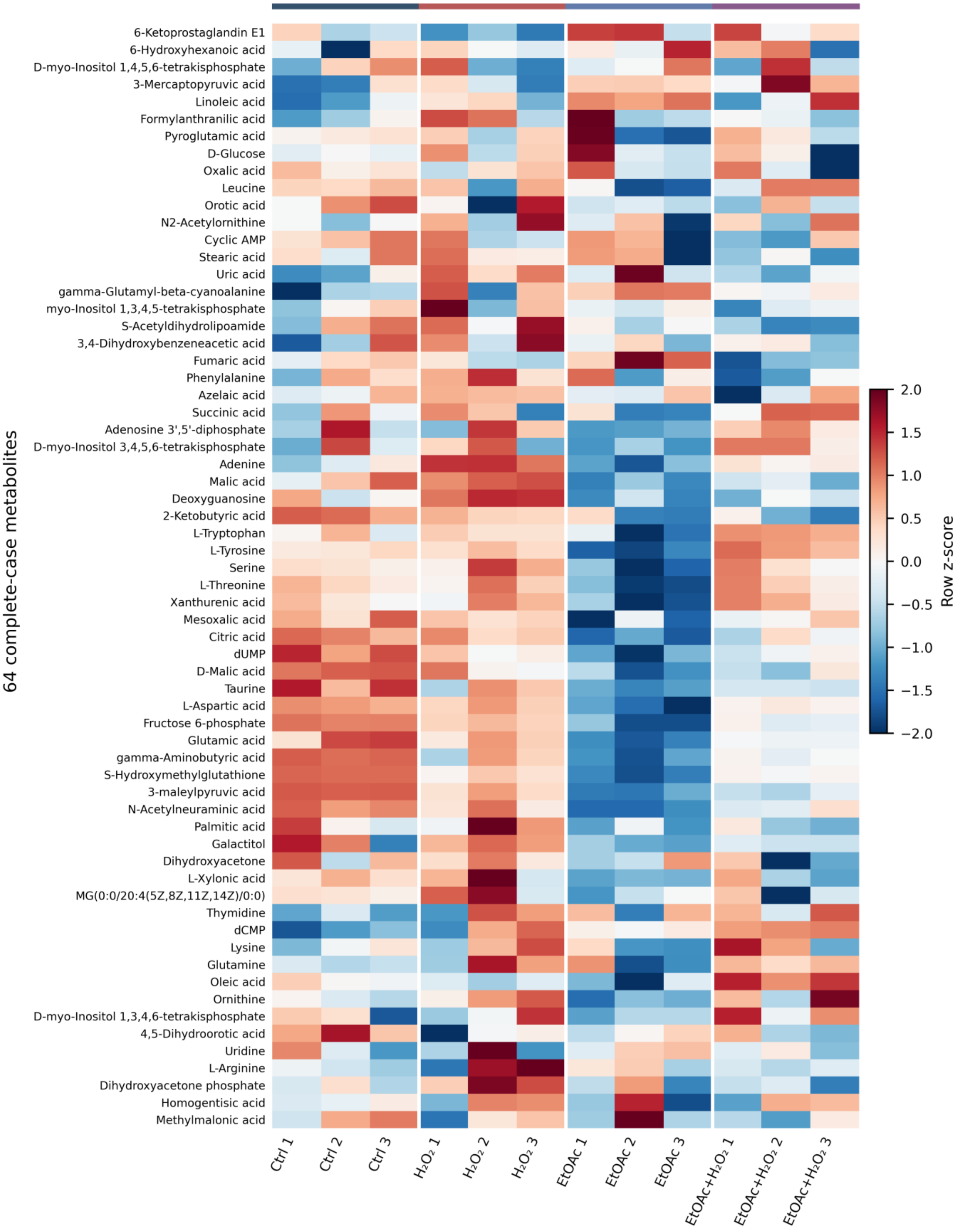

Figure S2. Descriptive heatmap of Control, H_2O_2, EtOAc Fr., EtOAc Fr. + H_2O_2. The 64 complete-case metabolites were clustered using average linkage and Euclidean distance after $\log_2$ transformation and row-wise z-scoring across the displayed samples. Color saturation corresponds to z-scores ≤ -2 or $\geq +2$. Each column represents one biological replicate after averaging analytical injections (n = 3 per group); colors and clusters do not show statistical significance.

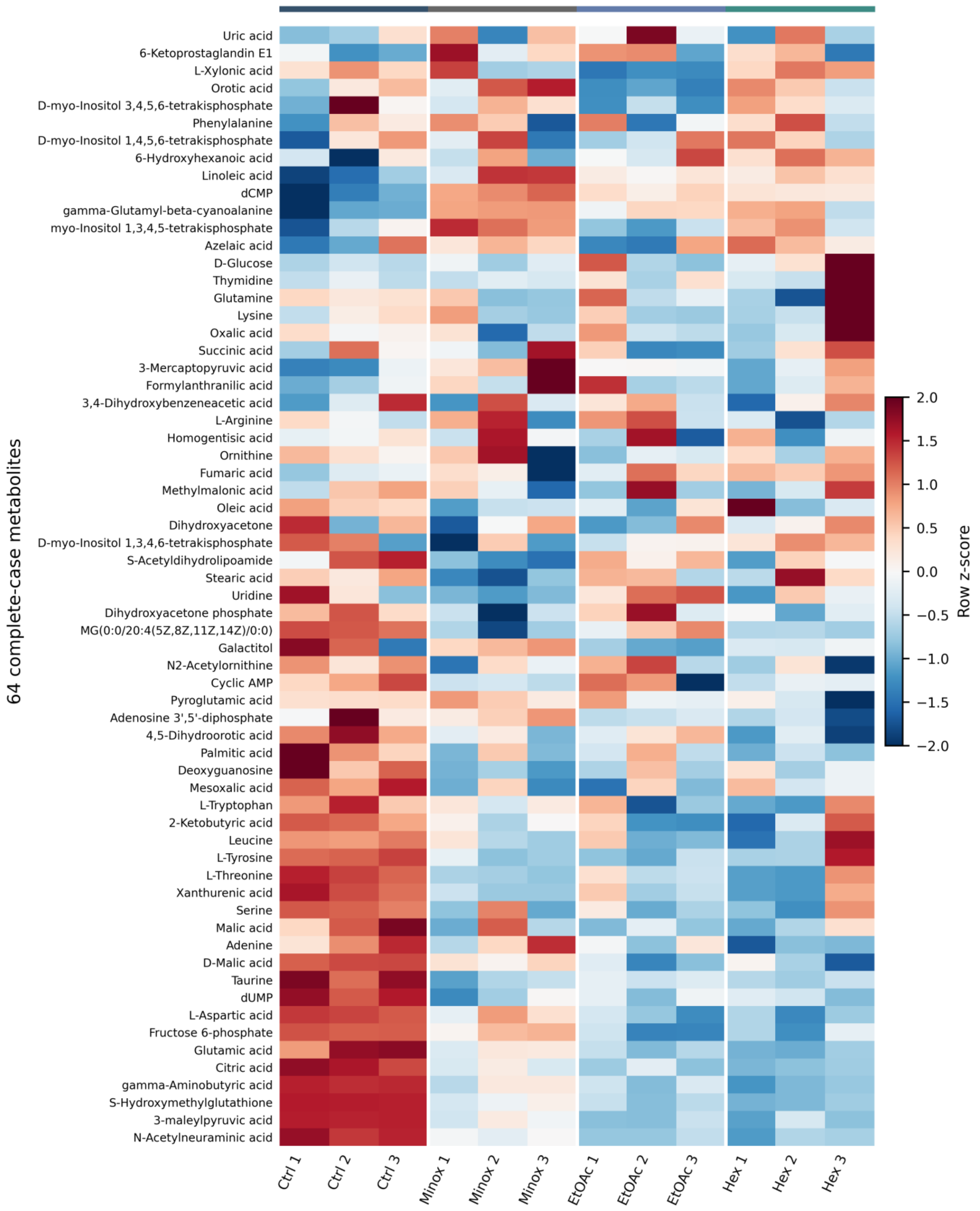

Figure S3. Descriptive heatmap of Control, Minoxidil, EtOAc Fr., n-Hexane Fr. The 64 complete-case metabolites were clustered using average linkage and Euclidean distance after $\log_2$ transformation and row-wise z-scoring across the displayed samples. Color saturation corresponds to z-scores ≤ -2 or $\geq +2$. Each column represents one biological replicate after averaging analytical injections (n = 3 per group); colors and clusters do not show statistical significance.

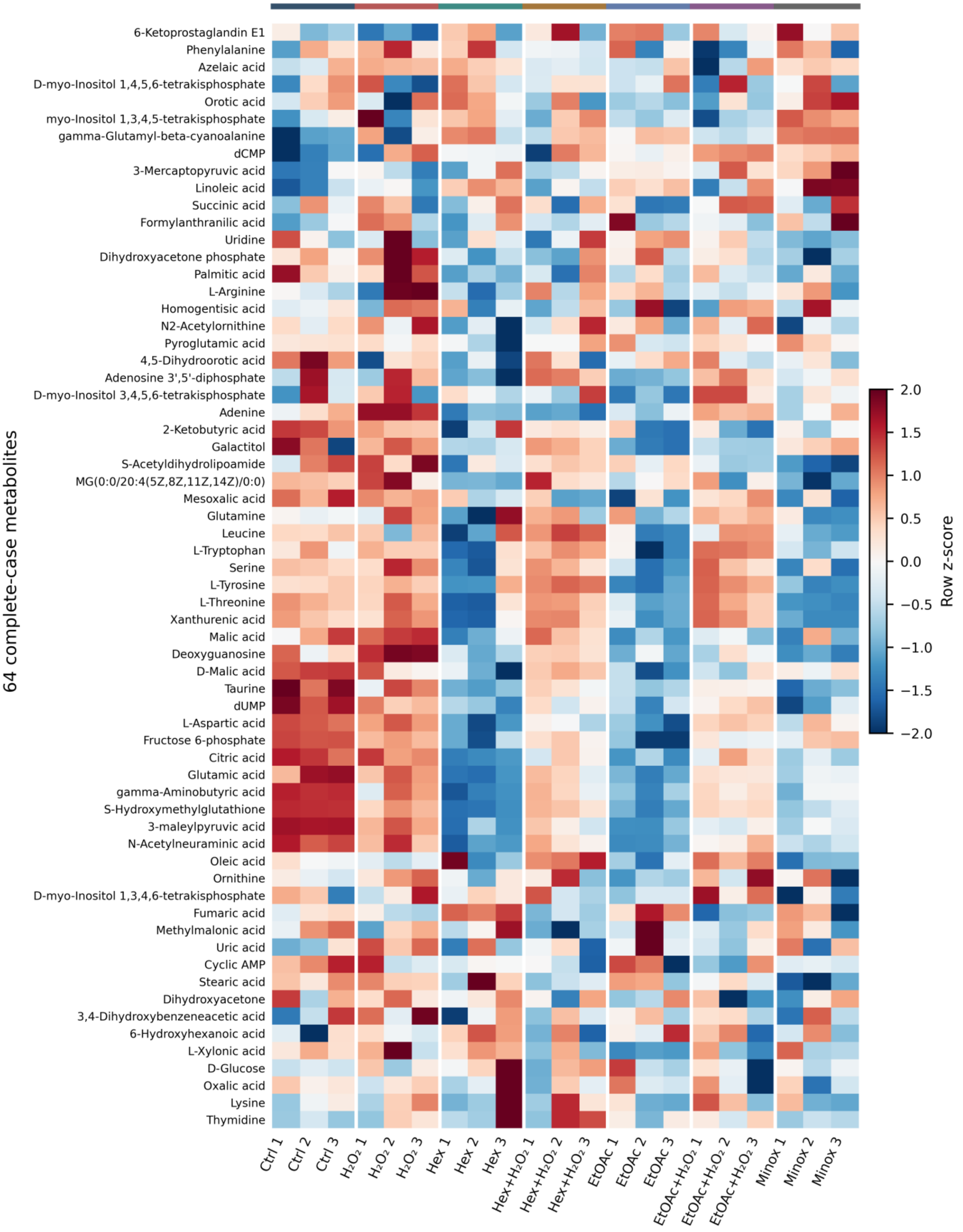

Figure S4. Descriptive heatmap of Control, H_2O_2, n-Hexane Fr., n-Hexane Fr. + H_2O_2, EtOAc Fr., EtOAc Fr. + H_2O_2, Minoxidil. The 64 complete-case metabolites were clustered using average linkage and Euclidean distance after $\log_2$ transformation and row-wise z-scoring across the displayed samples. Color saturation corresponds to z-scores ≤ -2 or $\geq +2$. Each column represents one biological replicate after averaging analytical injections (n = 3 per group); colors and clusters do not show statistical significance.
